# SLC25A42 promotes gastric cancer growth by conferring ferroptosis resistance through enhancing CPT2-mediated fatty acid oxidation

**DOI:** 10.1038/s41419-025-07644-7

**Published:** 2025-04-17

**Authors:** Haoying Wang, Weijia Dou, Mengxiao Liu, Weifang Wang, Ying Yang, Jibin Li, Zhenxiong Liu, Nan Wang

**Affiliations:** 1 Department of Gastroenterology, Tangdu Hospital, The Air Force Medical University, Xi’an, China; 2 Department of Gastroenterology, Xijing Hospital, The Air Force Medical University, Xi’an, China; 3 State Key Laboratory of Holistic Integrative Management of Gastrointestinal Cancers and Department of Physiology and Pathophysiology, The Air Force Medical University, Xi’an, China; 4 Department of General Surgery, Tangdu Hospital, The Air Force Medical University, Xi’an, China

**Keywords:** Gastric cancer, Gastric cancer

## Abstract

Accumulating evidence has shown that the dysfunction of mitochondria, the multifunctional organelles in various cellular processes, is a pivotal event in the development of various diseases, including human cancers. Solute Carrier Family 25 Member 42 (SLC25A42) is a mitochondrial protein governing the transport of coenzyme A (CoA). However, the biological roles of SLC25A42 in human cancers are still unexplored. Here we uncovered that SLC25A42 is upregulated and correlated with a worse prognosis in GC patients. SLC25A42 promotes the proliferation of gastric cancer (GC) cells while suppresses apoptosis in vitro and in vivo. Mechanistically, SLC25A42 promotes the growth and inhibits apoptosis of GC cells by reprograming lipid metabolism. On the one hand, SLC25A42 enhances fatty acid oxidation-mediated mitochondrial respiration to provide energy for cell survival. On the other hand, SLC25A42 decreases the levels of free fatty acids and ROS to inhibit ferroptosis. Moreover, we found that SLC25A42 reprograms lipid metabolism in GC cells by upregulating the acetylation and thus the expression of CPT2. Collectively, our data reveal a critical oncogenic role of SLC25A42 in GCs and suggest that SLC25A42 represent a promising therapeutic target for GC.

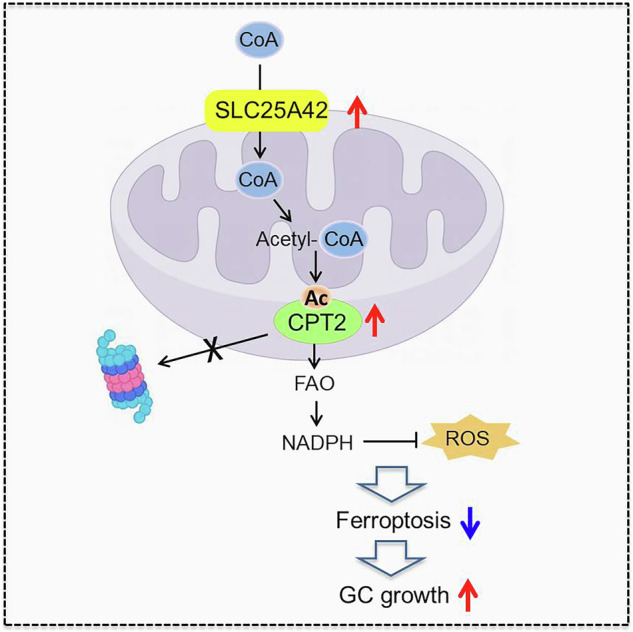

## Introduction

Gastric cancer (GC) is one of the deadly malignancies worldwide [1]. Despite advancements in surgical techniques, chemotherapy and radiotherapy, the prognosis of GC patients remains poor due to the absence of effective targeted therapies [2], highlighting the urgent need for further explorations of the key molecular factors driving the initiation and progression gastric cancer to unlock specific therapeutic strategy and target to improve the patient outcomes.

Mitochondria not only serve as the “powerhouse” of cells for energy production, but also act as multifunctional organelles in various critical cellular processes, including cell survival and cell death, production of reactive oxygen species (ROS), and maintenance of calcium equilibrium [3]. These essential functions of mitochondria support a wide array of cellular processes, including bioenergetics, biosynthetic, and oncogenic signaling pathways throughout the process of tumorigenesis [4]. Perturbations of mitochondrial have been frequently observed in various physiological disorders, including cancer [5, 6]. However, the factors leading to the abnormal mitochondrial function in cancer cells remain not fully elucidated.

Solute carrier family 25 (SLC25) is the largest mitochondrial carrier family (consist 53 members), which transport solutes across the inner membrane of mitochondria [7]. Among these carriers, SLC25A42 plays a crucial role in the transport of mitochondrial coenzyme A (CoA) [8, 9], which is an essential cofactor in numerous biochemical reactions, including the tricarboxylic acid cycle, nutrient oxidation, histone acetylation, and synthesis of lipids [10]. Mutations in SLC25A42 have been associated with mitochondrial myopathy [11] and encephalomyopathy [12] in humans, highlighting the importance of SLC25A42 in normal physiological functions. However, the biological roles of SLC25A42 in human cancer progression are still unexplored.

This study focused on the roles and mechanisms of SLC25A42 in the metabolism and malignant properties in gastric cancer cells. We found that SLC25A42 is upregulated and correlated with a worse prognosis in GC patients. SLC25A42 promotes the growth of gastric cancer (GC) cells while suppresses ferroptosis by reprograming lipid metabolism through elevating the acetylation and thus expression of CPT2, implying that SLC25A42 could serve as a potential therapeutic strategy for the treatment of this malignancy.

## Materials and methods

### Clinical samples

Clinical gastric cancer (GC) samples (*n* = 450) were collected at the Tangdu hospital of the Air Force Medical University from GC patients with informed consent from each one. The collection protocol was in accordance with the principles of the Declaration of Helsinki and approved by the Institute Research Ethics Committees at the Air Force Medical University.

### Cell lines

GC cell lines (SNU-638, MKN-1, HGC-27, MKN-45, SNU-216) and a normal gastric cell line (GES-1) were cultured at 37 °C in a humidified incubator using RPMI-1640 or DMEM medium containing 10% FBS. These cell lines were all confirmed to be mycoplasma-free and authenticated by the short tandem repeat (STR) profiling before our investigations.

### Quantitative PCR (qPCR)

RNA in fresh-frozen GC tissues and cultured cells was separated using the RNA Isolation Kit (Beyotime). RNA quality and concentration were then assessed with a Nanodrop spectrophotometer. After that, cDNA was synthesized with the PrimeScript RT Reagent Kit (TaKaRa). The primers listed in the Table S1 and SYBR Premix Ex Taq II (TaKaRa) were used for PCR reaction. Results were analyzed by the 2^−ΔΔCt^ method.

### Western blot analysis

Protein was first extracted from GC tissues or cultured GC cell lines. Protein was then quantified by BCA method, separated on SDS-PAGE gels, and transferred to PVDF membranes. The membranes were then incubated with 5% non-fat milk in TBST at room temperature (1 h) and probed with primary antibodies (Table S2) diluted in blocking buffer overnight at 4 °C. After washing twice for 10 min, HRP-conjugated secondary antibodies were added and incubated for 1 h at 26 °C. The results were obtained using the ECL detection system, and the relative expressions of target proteins were assessed according to β-actin levels.

### Immunohistochemistry (IHC)

Tissue sections from GC patients or nude mice were treated with hot citrate buffer, followed by blocking with 5% BSA. Primary antibodies (shown in Table S2) and HRP-conjugated secondary antibodies were then added to the sections and incubated overnight or two hours, respectively. The staining results were scored by evaluating both the intensity (strong staining was scored as 3; moderate staining was scored as 2; weak staining was scored as 1; no staining was scored as 0) and the expression extent (less than 25% was scored as 1; 25–50% was scored as 2; 50–75% was scored as 3; over 75% was scored as 4).

### Gene expression silencing and overexpression

Gene expression modulation was performed using shRNA constructs silencing of target genes and expression vectors for overexpressing. Lipofectamine 2000 reagent was used for transfections. After 48 h, the efficiencies of knockdown or overexpression were verified by qPCR and Western blotting analysis.

### CCK-8 cell proliferation assay

Cells were seeded in 96-well plates (5000 cells/well) and Cell Counting Kit-8 (CCK-8) was undertaken to assess cell proliferation. After 1, 2, 3 or 4 days of incubation, 10 µL of CCK-8 reagent (Beytime) was added to the cells. Two hours later at, the results were obtained by measuring the absorbance (450 nm).

### EdU assay

Cell cycle progression-associated proliferation was also assessed by the EdU staining assay. GC cells in 6-well plates were incubated with EdU (10 µM) at 35 °C for one hour. Then, cells were fixed by paraformaldehyde and permeabilized with 0.5% Triton X-100. DAPI was used for nuclei counterstaining. The results were assessed under a confocal microscope.

### Colony formation assay

GC cell lines (200 cells/well) were plated to 6-well plates. After about 10–14 days, cells were washed by PBS and fixed using paraformaldehyde. Then, crystal violet was used for staining. Finally, colonies in each well of the 6-well-plates were counted.

### Scratch wound healing assay

Cells were cultured in 6-well plates and a scratch was created with a pipette tip. After washing with PBS, serum-free medium was added and images of the scratch area were captured at 0 and 24 h under a microscope. Relative migration abilities were assessed as the percentage of wound closure.

### Transwell assay

For transwell invasion assay, GC cells in serum-free media were added to the upper chamber (8 µm pore size) of the transwell. Twenty four hours post cells incubation, invaded cells adhered to the lower surface were fixed with paraformaldehyde, stained and counted under a microscope.

### Subcutaneous tumor growth assay in nude mice

For in vivo tumorigenicity studies, GC cells (5 million) were injected subcutaneously into the 4-week-old male BALB/c nude mice (six mice per group, randomly assigned to different groups). Animals were housed in a controlled environment and received care in accordance with the guidelines from the Institutional Animal Care and Use Committee of the Air Force Medical University. Tumor size was monitored by caliper measurements weekly. The mice were euthanized until they reached ethical endpoints (4 weeks). Additionally, BrdU-Red DNA Fragmentation (TUNEL) Assay Kit (ab66110) was used for evaluation of cell apoptosis in tumor tissues from the nude mice.

### Tail vein injection for lung metastasis

To evaluate the metastatic potential of GC cells in vivo, 5 million cells were injected into the tail vein of the nude mice (six mice per group, randomly assigned to different groups). Five weeks post-cell injection, the mice were euthanized, and their lung tissues were harvested for histological H&E analysis to quantify the number of metastatic tumor nodules. Animals were housed in a controlled environment and received care in accordance with the guidelines provided by the Institutional Animal Care and Use Committee of the Air Force Medical University.

### Cellular reactive oxygen species (ROS) level

Cellular ROS levels were quantified with the fluorescent dye DCFDA (Beytime). Firstly, GC cells plated in confocal dish were incubated with 10 µM DCFDA at 37 °C for 25 min. After washing 3 times with PBS, the fluorescence staining was assessed using a confocal microscopy.

### Flow cytometric analysis of cell apoptosis and cell death

Cell apoptosis and death were assessed using Annexin V/PI staining kit (Beytime). Cells were incubated in binding buffer and Annexin V-FITC and PI solution in the dark 15 min at 28 °C. Flow cytometer was used for results analysis. Cells were considered as apoptotic if Annexin V-positive and PI-negative, while cell death was considered as Annexin V-positive and PI-positive.

### Flow cytometric analysis of cell cycle

To analyze the cell cycle distribution, GC cells were fixed in 70% ethanol overnight at 4 °C and subsequently stained with propidium iodide (PI) solution containing RNase A for 30 min. Flow cytometer was used to determine the percentages of cells in each phase of the cell cycle under different treatment conditions.

### Detection of cellular lipid content

Cellular lipid contents triglycerides, phospholipids, and cholesterol were quantified using commercial kits from Abcam. Cells were washed by PBS and suspended in lysis buffer. Absorbance measurements were taken at specified wavelengths following the manufacturer’s instructions for each assay. Data were normalized to the number of cells.

### Lipid peroxidation assay

Lipid peroxidation levels were determined using the BODIPY-C11 kit (Thermo Fisher). Cells were plated in confocal dish and cultured overnight. Then, BODIPY-C11 reagent was added to cells and incubated for 30 min at 37 °C in the dark. After washing 3 times, the results were photographed and analyzed using a confocal microscopy.

### Measurement of intracellular Fe^2+^ levels

Intracellular levels of Fe^2+^ were measured using the FerroOrange fluorescent dye (Dojindo). Cells plated in confocal dish were incubated with FerroOrange reagent 30 mins at 37 °C in the dark. After washing 3 times, the staining results were photographed and analyzed using a confocal microscopy.

### Mitochondrial fluorescence staining

To assess mitochondrial morphology, GC cells were stained with the mitochondrial-specific fluorescent probe MitoTracker (Thermo Fisher). Cells were firstly seeded in confocal dish and cultured overnight. After washing with PBS, MitoTracker solution was added and cultured for 30 mins at 37 °C in the dark. Following washing with PBS to remove excess dye, the fluorescence images were obtained using a confocal microscope.

### Mitochondrial membrane potential

To assess mitochondrial membrane potential, GC cells seeded in confocal plates were stained with 2 µM JC-1 dye (Invitrogen) for 20 min at 37 °C. Following washing with PBS 3 times, the results were analyzed using a confocal microscopy to determine the red/green fluorescence ratio.

### Cellular ATP content

The content of cellular ATP was measured using an ATP detection kit (Invitrogen) according to the manufacturer’s instructions. GC cells were lysed and the lysate was mixed with ATP detection solution. Luminescence was quantified using a luminometer and ATP levels were normalized to protein concentration.

### Oxygen consumption rate (OCR) and extracellular acidification rate (ECAR) analysis

A seahorse XF analyzer was used to determine the oxygen consumption rate (OCR) and extracellular acidification rate (ECAR) to evaluate cellular respiration and glycolytic metabolic capacities. For OCR analysis, GC cells were seeded onto XF24 plates and analyzed for basal OCR following the treatments with oligomycin, FCCP, and antimycin A. For measurement of ECAR, GC cells were seeded onto XF24 plates and baseline ECAR was recorded, which was followed by the treatments with glucose, oligomycin, and 2-deoxyglucose (2-DG).

### Statistical analysis

Statistical analyses were conducted using the SPSS 14.0 software. Data were expressed as mean ± standard deviation (SD) from at least three replications to ensure reliability and reproducibility. Comparisons were conducted using one-way ANOVA with post-hoc tests for multiple groups or using Student’s *t*-test for two groups. *P* value less than 0.05 were considered as statistically significant. The in vitro experiments were repeated at least three times.

## Results

### SLC25A42 is upregulated and correlated with a worse prognosis in patients with gastric cancer

**Fig. 1:**
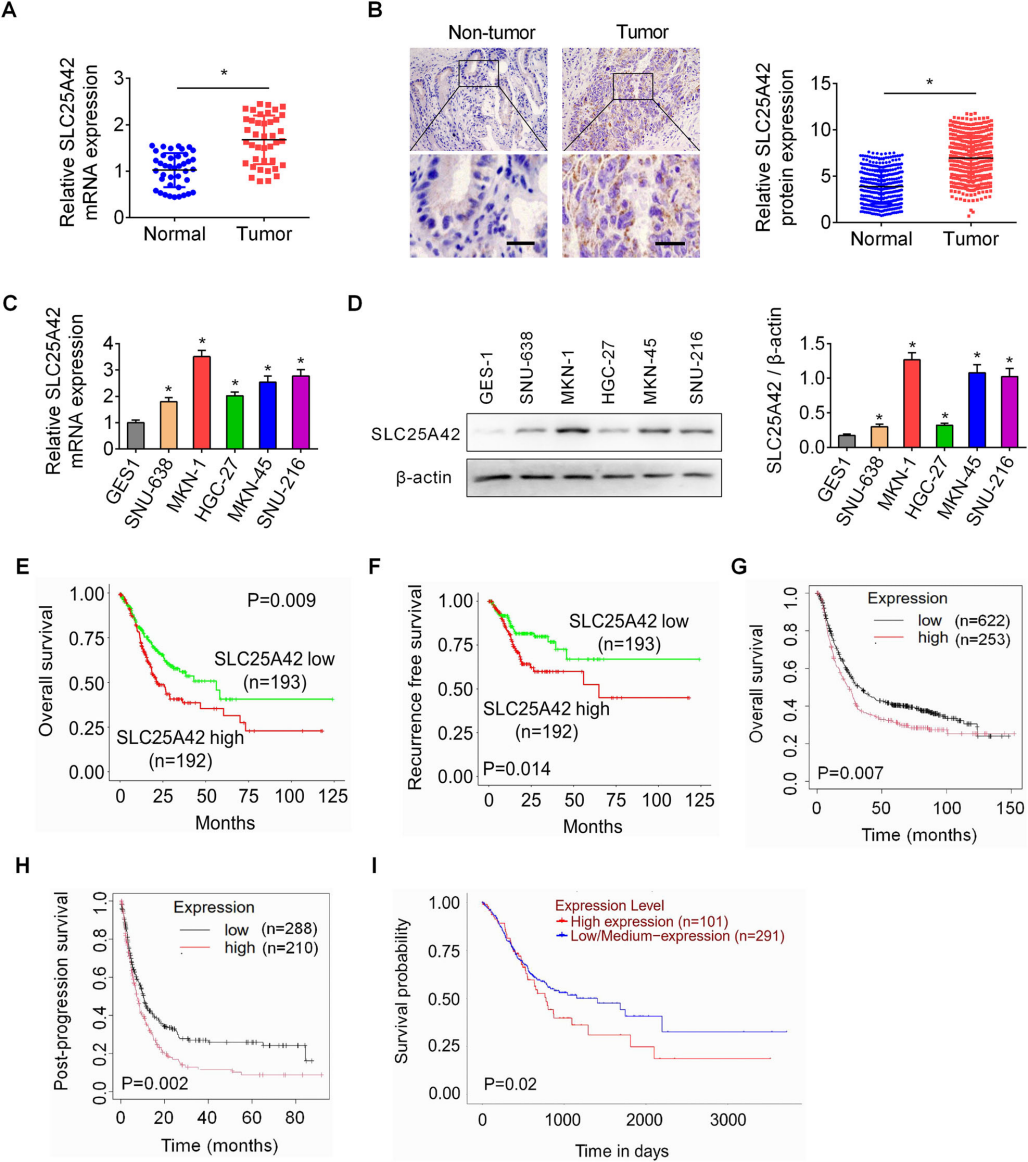
SLC25A42 is upregulated and correlates with worse prognosis in patients with gastric cancer. The detection of SLC25A42 expression was carried out through qPCR (**A**, *n* = 40) and IHC (**B**, *n* = 385) analysis in paired GC and matched para-cancerous tissues. Scale bar, 20 μm. **C**, **D** CSM5 expression was also evaluated in a serial of widely used human GC cell lines (SNU-638, MKN-1, HGC-27, MKN-45, SNU-216) and a normal control gastric cell line (GES-1) through qPCR and Western blot analysis. Kaplan–Meier analysis was performed to compare the survival (**E**, overall survival; **F**, disease free survival) of GC patients with low-SLC25A42 and high-SLC25A42 levels. Overall survival (**G**) and post-progression survival (**H**) analysis of the gene chip expression database using the online Kaplan-Meier Plotter. **I** Overall survival analysis of TCGA database using the online UALCAN. *P < 0.05.

To assess the expression of SLC25A42 in GC, we initially examined SLC25A42 expression utilizing qPCR and immunohistochemistry (IHC) assays in GC and para-cancerous tissues (*n* = 40 for qPCR assay; *n* = 385 for IHC assay). A notable elevation in SLC25A42 expression was observed within GC tissues as compared with corresponding normal gastric tissues (Fig. 1A, B). Additionally, GC patients exhibiting larger tumor size and advanced stages were associated with markedly increased SLC25A42 expression (Table S3). Moreover, SLC25A42 expression was also found to be higher in a serial of human gastric cancer cell lines (SNU-638, MKN-1, HGC-27, MKN-45, SNU-216) in comparison to primary human normal gastric cells (GES-1) (Fig. 1C, D).

Subsequently, based on the IHC staining scores, the 385 GC patients were categorized into low-SLC25A42 (*n* = 193) and high-SLC25A42 (*n* = 192) groups. Kaplan-Meier analyses revealed that patients with low-SLC25A42 levels had more favorable overall survival (OS) and disease free survival (DFS) compared to those with higher-SLC25A42 levels (Fig. 1E, F). In agreement with this, survival analysis of the public gene chip expression database using the online Kaplan–Meier Plotter [13] also indicated that upregulation of SLC25A42 was correlated with worse survival for patients with GC (Fig. 1G, H). Similarly, analysis of the public TCGA database using the online UALCAN indicated that GC patients expressing high SLC25A42 had clearly poorer survival than those expressing low SLC25A42, although the difference was not significant (Fig. 1I).

### SLC25A42 promotes the proliferation while suppresses cell death of gastric cancer (GC) cells in vitro

**Fig. 2:**
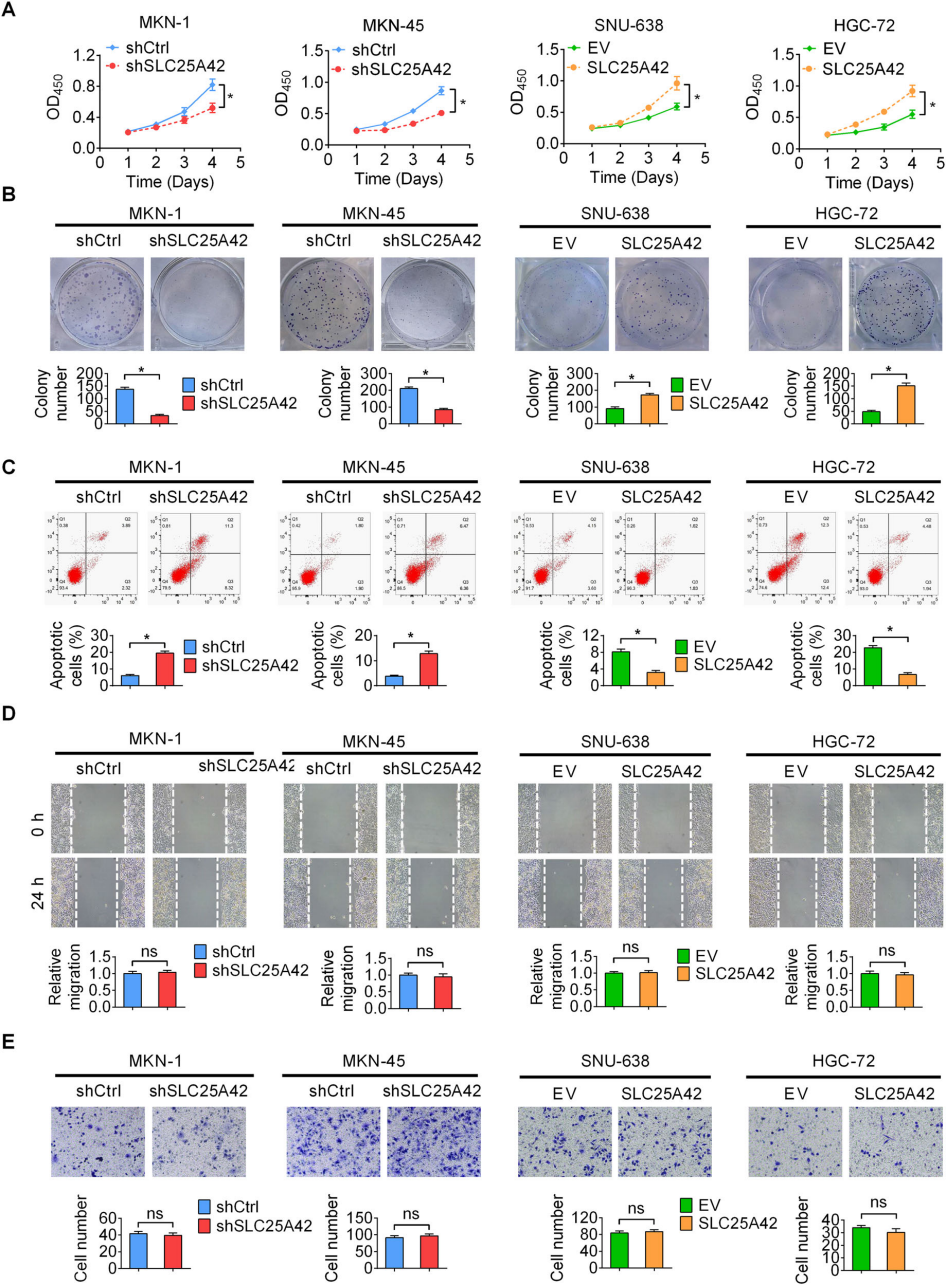
SLC25A42 promotes the proliferation while suppresses cell death of gastric cancer (GC) cells in vitro. CCK8 (**A**) and colony formation (**B**) elevations for the proliferative potential of gastric cancer (GC) cells with SLC25A42 knockdown or overexpression. **C** Flow cytometry was utilized to investigate the rates of cell death of GC cells with different SLC25A42 levels (**D**) The wound healing assay was conducted to compare the migratory abilities of GC cells. **E** The transwell assay was used to compare the invasive capabilities of GC cells. *P < 0.05; ns, not significant.

Based on the expression levels of SLC25A42 in GC cell lines, we chose MKN-1 and MKN-45 for SLC25A42 knockdown, and SNU-638 and HGC-27 for SLC25A42 overexpression, subsequently investigating the effect of SLC25A42 on cellular behavior in vitro. The efficiencies of silencing or forced expression were tested and confirmed by qPCR (Fig. S1A) and Western blot analysis (Fig. S1B). Results from CCK8 and colony formation assays demonstrated that the knockdown of SLC25A42 led to a significant reduction in the proliferation of MKN-1 and MKN-45 cells, whereas the overexpression of SLC25A42 resulted in increased proliferation of SNU-638 and HGC-27 cells (Fig. 2A, B). Flow cytometric evaluation of cell cycle and apoptosis revealed that SLC25A42 knockdown markedly increased cell death, while SLC25A42 overexpression decreased cell death in SNU-638 and HGC-27 cells (Fig. 2C). However, both SLC25A42 knockdown and overexpression had no notable effect on cell cycle distribution in GC cells (Fig. S1C), which was further verified by EdU assay (Fig. S1D). We also evaluated the effects of SLC25A42 on the migratory and invasive characteristics of GC cells. No significant changes in the migration and invasion capabilities of GC cells were observed when SLC25A42 was either knocked-down or over-expressed (Fig. 2D, E). These data suggest that SLC25A42 promotes the proliferation while suppresses cell death of gastric cancer (GC) cells in *vitro*.

### SLC25A42 promotes in vivo GC tumor growth

**Fig. 3:**
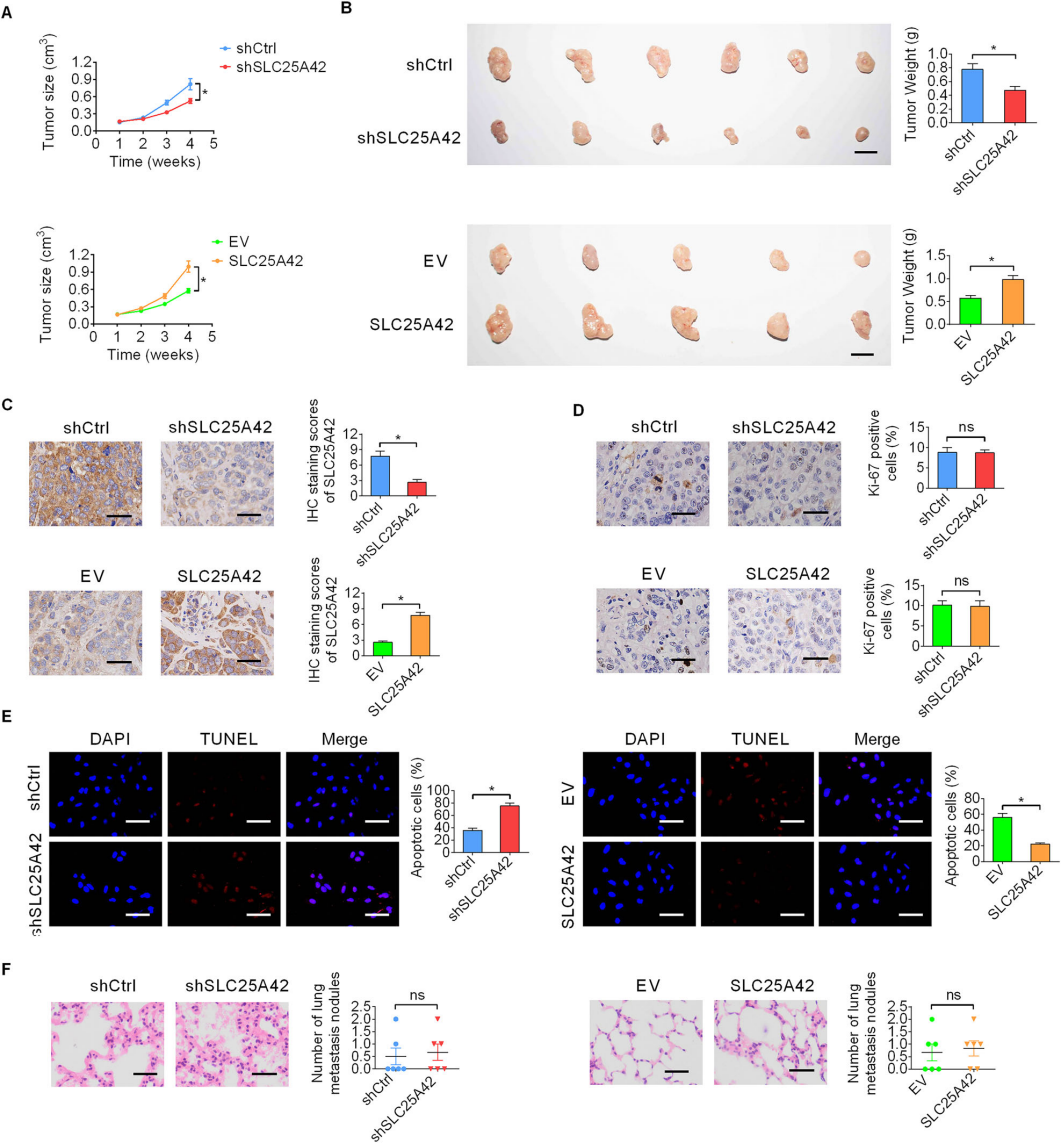
SLC25A42 promotes in vivo GC tumor growth. **A** Subcutaneous tumor models were established using SLC25A42 silencing MKN-1 cells or overexpressing SNU-638 cells and their corresponding control cells (*n* = 6; Scale bar, 1 cm). The images of excised subcutaneous tumors were shown. **B** The weights of the tumors were compared in indicated groups. The expression levels of SLC25A42 (**C**) and Ki-67 (**D**) in the tissues from subcutaneous nude mice models were assessed by IHC assay. Scale bar, 20 μm. **E** The occurrence of apoptosis-positive cells in the tissues from subcutaneous nude mice models was assessed by TUNEL assay. Scale bar, 20 μm. **F** The number of nodules in the lungs from indicated groups was compared by the H&E analysis. Scale bar, 10 μm. *P < 0.05; ns, not significant.

To clarify the biological functions of SLC25A42 in vivo, we established subcutaneous tumor models in the nude mice. Measurements of the growth curves and the final weights of the tumors showed that the growth of GC was significantly inhibited by SLC25A42 silencing in MKN-1 cells, while promoted by overexpression of SLC25A42 in SNU-638 cells (Fig. 3A, B). The subcutaneous tumors were then fixed, embedded, and sectioned for additional IHC and TUNEL analysis. Evaluation of SLC25A42 and Ki67 expressions by IHC analysis indicated that knockdown or overexpression of SLC25A42 (Fig. 3C) had no notable effect on the percentage of proliferating cells (Fig. 3D), while the TUNEL assay showed negatively regulated cell apoptosis by SLC25A42 in subcutaneous GC tissues (Fig. 3E). Additionally, in line with the results of in vitro cell migration and invasion assays, the in vivo lung metastases assessed by H&E staining analysis showed that the number of lung metastases did not change significantly either when SLC25A42 was knocked-down or overexpressed (Fig. 3F). The above data indicate that knockdown of SLC25A42 suppresses the in vivo growth of GC.

### SLC25A42 inhibits ferroptosis in GC cells

**Fig. 4:**
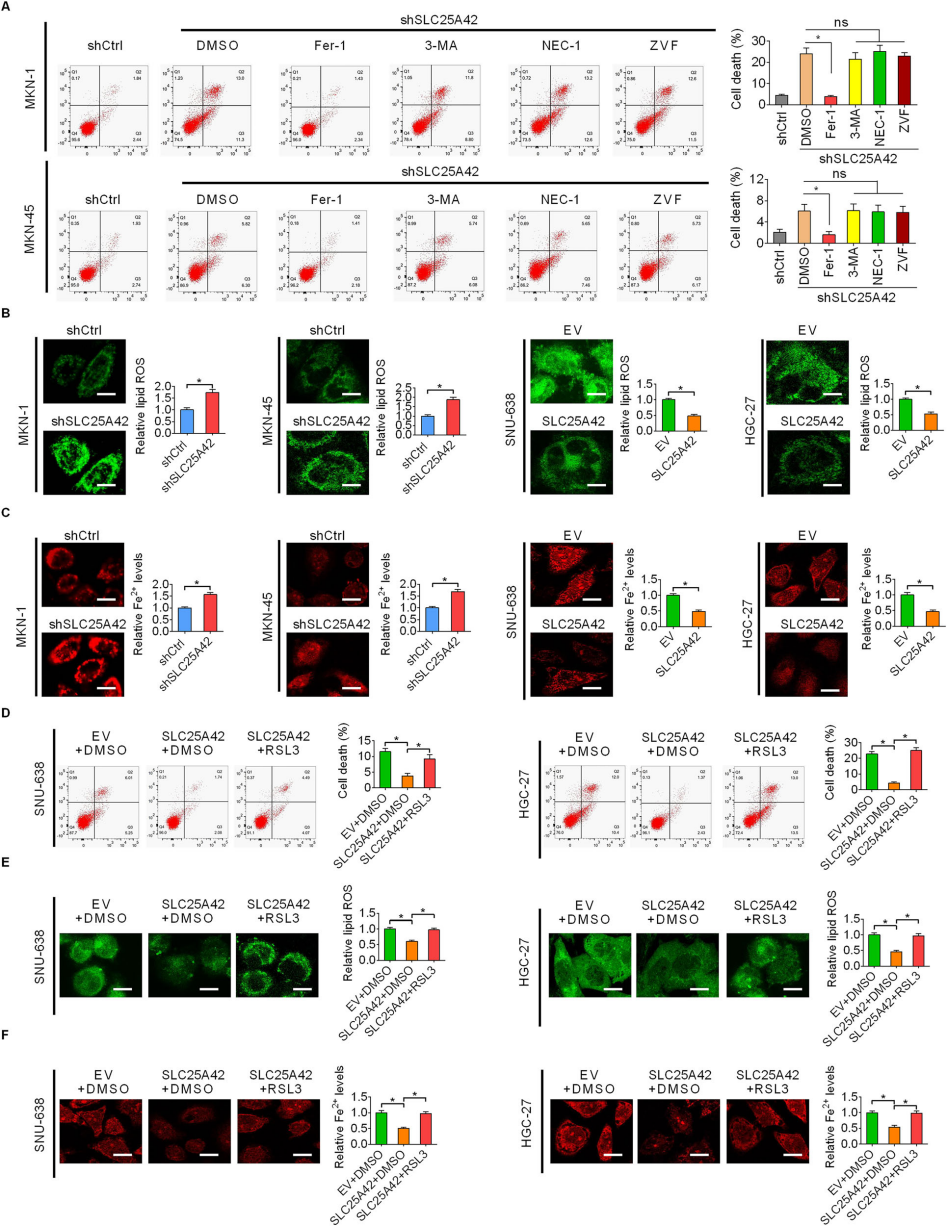
SLC25A42 inhibits ferroptosis in GC cells. **A** Flow cytometry was utilized to investigate the rates of cell death of GC cells treated with specific inhibitors targeting different types of programmed cell death. 3-Methyladenine (3-MA) targeting autophagy, Z-VAD-FMK (ZVF) targeting apoptosis, Necrostatin-1 (NEC-1) targeting necroptosis. The levels of lipid peroxidation (**B**) and intracellular Fe^2+^ (**C**) were assessed in GC cells exhibiting either SLC25A42 knockdown or overexpression. Scale bar, 10 μm. **D** IHC staining of 4-hydroxy-2-noneal (4-HNE) in xenograft tumor tissues developed from SLC25A42 knockdown or control MKN-1 cells. **F** Flow cytometry was utilized to investigate the rates of apoptosis of GC cells. The levels of lipid peroxidation (**E**) and intracellular Fe^2+^ (**F**) were assessed in GC cells treated with the ferroptosis inducer RSL3. Scale bar, 10 μm. *P < 0.05.

During recent years, several different types of programmed cell death, including apoptosis, autophagy, ferroptosis, and necroptosis, have been recognized. To investigate which type of programmed cell death was suppressed by SLC25A42, SLC25A42-knockdown MKN-1 and MKN-45 cells were treated with specific inhibitors targeting these programmed cell death types, respectively. As indicated in Fig. 4A, cell death enhanced by SLC25 A42 knockdown was notably reversed by ferroptosis inhibitor Fer-1 treatment but not by inhibitors targeting other types of cell death, suggesting that knockdown of SLC25A42 induces ferroptosis in GC cells. Additionally, evaluations of major characteristics of ferroptosis revealed that the levels of both lipid peroxidation and Fe^2+^ were elevated by SLC25A42 knockdown, while reduced upon SLC25A42 overexpression in GC cells (Fig. 4B, C). Consistently, IHC staining in xenograft tumor tissues from nude mice models indicated increased level of 4-hydroxy-2-noneal (4-HNE), which is a lipid peroxidation marker, when SLC25A42 was silenced (Fig. S2). Moreover, we found that levels of cell death, lipid peroxidation, and Fe^2+^ content decreased by SLC25A42 could be reversed by RSL3 treatment-induced ferroptosis (Fig. 4D–F). Collectively, these results indicate that SLC25A42 plays a crucial role in ferroptosis inhibition in GC cells.

### SLC25A42 enhances fatty acid oxidation (FAO)-supported mitochondrial respiratory activity in GC cells

**Fig. 5:**
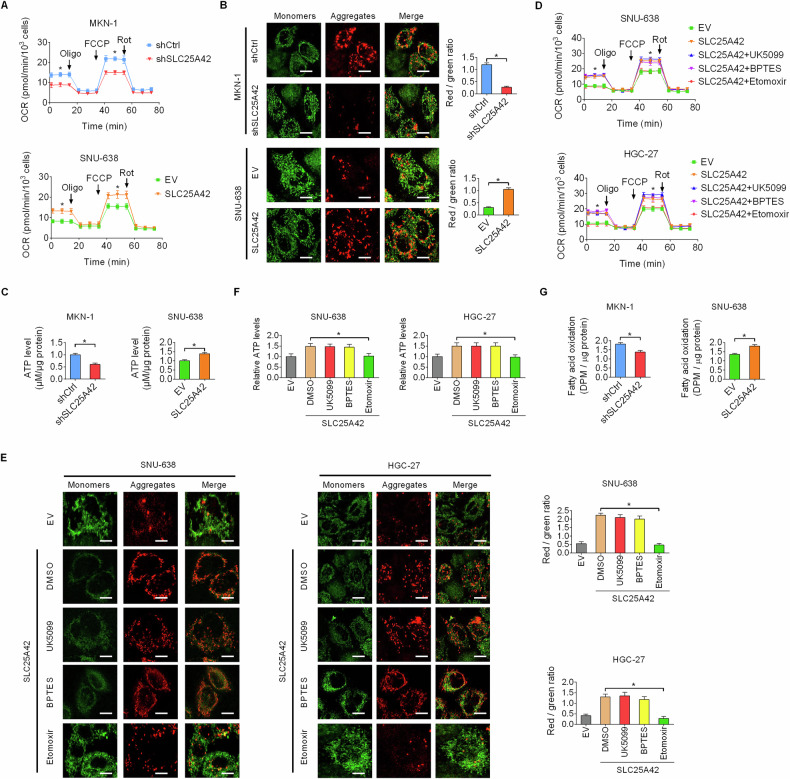
SLC25A42 enhances fatty acid oxidation (FAO)-supported mitochondrial respiratory activity in GC cells. Mitochondrial oxygen consumption rate (OCR) (**A**), membrane potential (**B**; Scale bar, 5 μm), and intracellular levels of ATP (**C**) were evaluated in SLC25A42-silencing MKN-1 and SLC25A42-overexpresing SNU-638 cells. Mitochondrial OCR (**D**), membrane potential (**E**; Scale bar, 5 μm), and intracellular levels of ATP (**F**) were evaluated in SLC25A42-silencing MKN-1 and MKN-45 cells treated with inhibitors targeting glucose, fatty acid, and glutamine. **G** The rate of FAO was assessed using oleic acid (^3^H-labeled) as the trace in SLC25A42-silencing MKN-1 and SLC25A42-overexpresing SNU-638 cells. *P < 0.05.

Although SLC25A42 has been recognized as a mitochondria-localized protein by MitoCarta3.0 database [14], its expression in gastric cancer cells remains unknown. Using double immunofluorescence staining of SLC25A42 and mitochondrial in MKN-1 and MKN-45 cells, we confirmed that SLC25A42 is predominantly expressed in mitochondria of GC cells (Fig. S3A). Given that SLC25A42 is a mitochondrial transport carrier regulating the transport of coenzyme A (CoA), a cofactor playing essential roles in cell metabolism regulation, to explore the mechanism by which SLC25A42 regulates ferroptosis in GC cells, the effects of SLC25A42 on mitochondrial metabolism were thus explored. Knockdown of SLC25A42 led to obviously reduced mitochondrial oxygen consumption rate (OCR), membrane potential, and energy production in GC cells. Conversely, upregulation of SLC25A42 had the opposite impacts on these mitochondrial metabolic phenotypes in SNU-638 cells (Fig. 5A–C). Science glucose, fatty acid, and glutamine are the three major substrates of mitochondrial metabolism, we treated SLC25A42-overexpressing GC cells with inhibitors targeting these metabolic pathways to figure out the source of mitochondrial respiration enhanced by SLC25A42 in GC cells. We found that treatment with etomoxir, the inhibitor of fatty acid oxidation (FAO), markedly attenuated the activation of mitochondrial respiration activity by SLC25A42 overexpression, while inhibitors targeting glycolysis and glutaminolysis had no obvious impact on these mitochondrial metabolic phenotypes regulated by SLC25A42 (Fig. 5D–F). Further analysis using oleic acid (^3^H-labeled) as the trace verified that the FAO in GC cells was suppressed by SLC25A42 knockdown, while activated by SLC25A42 overexpression (Fig. 5G). These data suggest that SLC25A42 enhances mitochondrial respiratory activity by increasing fatty acid oxidation.

### SLC25A42 promotes GC cell proliferation and inhibits ferroptosis by activation of FAO

**Fig. 6:**
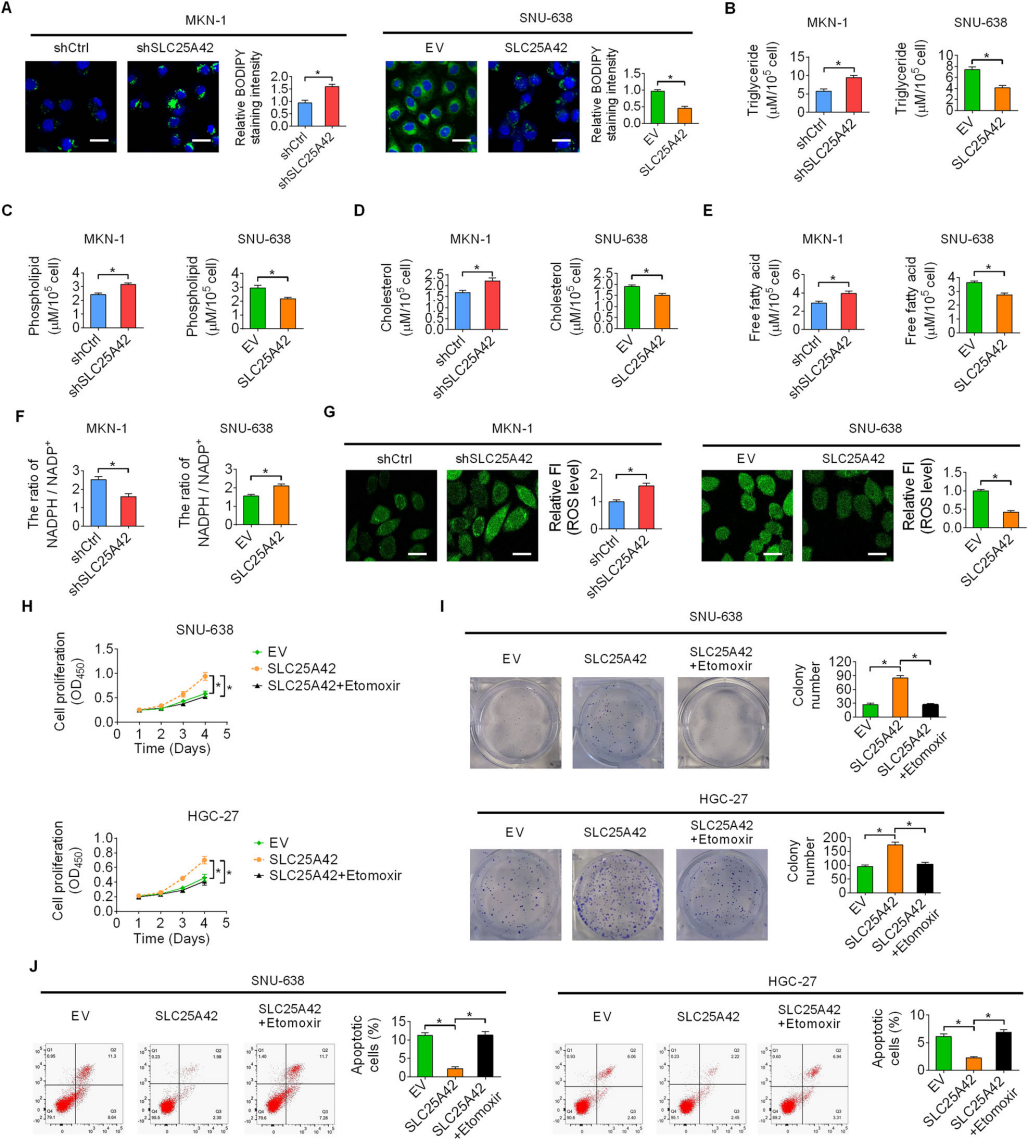
SLC25A42 promotes GC cell proliferation and inhibit ferroptosis by activation of FAO. **A** BODIPY (493/503) staining assay was used to assess lipid amount in SLC25A42-silencing MKN-1 and SLC25A42-overexpresing SNU-638 cells. Scale bar, 20 μm. The amounts of triglycerides (**B**), phospholipids (**C**), cholesterol (**D**), and free fatty acids (**E**) were determined in GC cells. The levels of NADPH (**F**) and ROS (**G**) were determined in GC cells. FI. fluorescence intensity. Scale bar, 20 μm. The CCK8 (**H**) and colony formation (**I**) assays for comparing the proliferative potential in SLC25A42-overexpresing SNU-638 cells treated with etomoxir. **J** Flow cytometry was utilized to investigate the rates of cell death in SLC25A42-overexpresing SNU-638 cells treated with etomoxir. *P < 0.05.

The involvement of SLC25A42 in the regulation of lipid metabolism was further verified by checking the lipid amount with BODIPY (493/503) staining in GC cells expressing either SLC25A42 knockdown or overexpression. We observed an increase of lipid content in the SLC25A42 knockdown MKN-1 cells as compared to the control MKN-1 cells, while less lipid content was observed in SLC25A42 overexpression SNU-638 cells as compared to the control (Fig. 6A). Consistently, the amounts of triglycerides, phospholipids, cholesterol and free fatty acids were increased in SLC25A42 knockdown MKN-1 cells while decreased in SLC25A42 overexpression SNU-638 cells (Fig. 6B–E). Given that SLC25A4 enhances FAO in GC cells, the levels of NADPH, which is the production of FAO, were thus determined. We discovered that the levels of NADPH were decreased upon SLC25A42 silencing, while increased when SLC25A42 was overexpressed (Fig. 6F). In agreement with the change in NADPH production, we found that the level of ROS was increased when SLC25A42 was knocked down, while decreased when SLC25A42 was overexpressed (Fig. 6G). Moreover, suppression of FAO by etomoxir clearly abolished the proliferation of GC cells promoted by upregulation of SLC25A42 (Fig. 6H, I), while rescued the ferroptosis of GC cells suppressed by upregulation of SLC25A42 (Fig. 6J), suggesting that SLC25A42 promotes GC cell proliferation and inhibit ferroptosis by activating FAO mainly through reducing energy production and ROS-mediated lipid peroxidation.

### SLC25A42 reprograms lipid metabolism in GC cells by upregulating the acetylation and thus the expression of CPT2

**Fig. 7:**
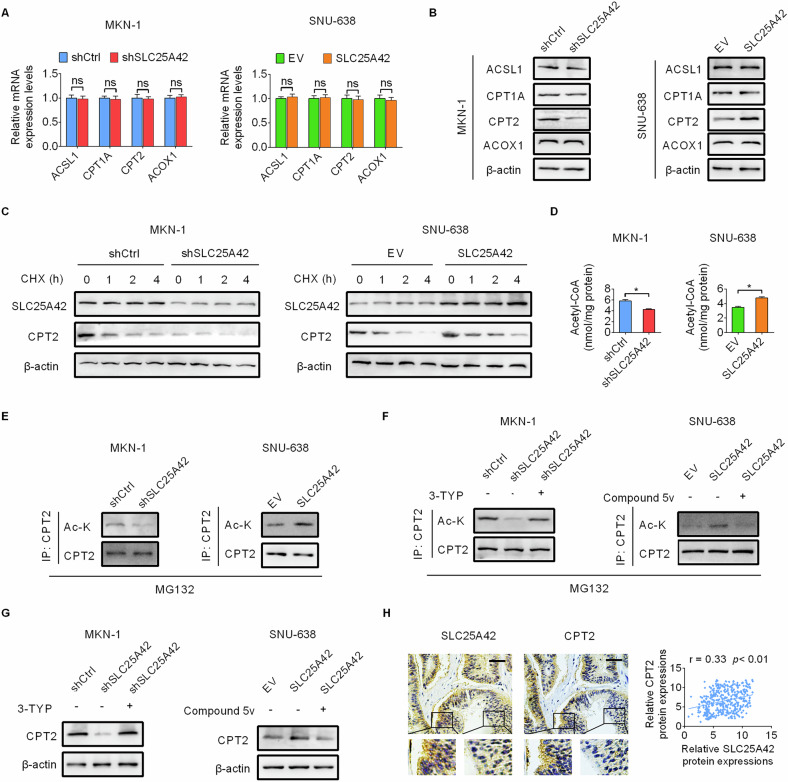
SLC25A42 reprograms lipid metabolism in GC cells by upregulating the acetylation and thus the expression of CPT2. The expressions of key FAO enzymes were detected by qPCR at mRNA level (**A**) and western blot at protein (**B**) level. **C** CPT2 expression was assessed by western blot assay in GC cells treated with CHX. The levels of acyl-CoA (**D**) and acetylation of CPT2 (**E**) were assessed by western blot assay in GC cells treated with MG132. **F** The acetylation of CPT2 was assessed by western blot assay in GC cells treated with SIRT1 inhibitor 3-TYP or activator Compound 5 v. **G** CPT2 expression was assessed by western blot assay in GC cells treated with SIRT1 inhibitor 3-TYP or activator Compound 5 v. **H** IHC staining images of CPT2 in GC tissues (left panel) and the correlation between the expressions of SLC25A42 and CPT2 were analyzed (right panel). Scale bar, 20 μm. *P < 0.05; ns, not significant.

We next explored how SLC25A42 reprograms the metabolism of FAO. We determined the impact of SLC25A42 on the expression of key FAO enzymes and revealed that the expression of CPT2 at protein level was clearly decreased by SLC25A42 knockdown while increased by SLC25A42 overexpression (Fig. 7A, B), suggesting a post-translational regulation of CPT2 expression by SLC25A42 in GC cells. Evaluation of the stability of CPT2 at protein level upon protein synthesis inhibition by CHX revealed that the degradation of CPT2 was clearly enhanced by SLC25A42 knockdown, while decreased by SLC25A42 overexpression (Fig. 7C). As a production of FAO and the major pool of cellular cofactor coenzyme A (CoA), acylated cofactor coenzyme (acyl-CoA) plays a crucial role in the post-translational acetylation of a variety of proteins to impact their stability and activity [15–17]. Having found that the stability of CPT2 was promoted by SLC25A42, we tested whether the acetylation of CPT2 was impacted by SLC25A42. The results showed that knockdown of SLC25A42 resulted in markedly decreased levels of acyl-CoA and the acetylation of CPT2, while the levels of acyl-CoA and acetylation of CPT2 were increased upon SLC25A42 overexpression (Fig. 7D, E). Additionally, we found that upregulation of CPT2 acetylation (Fig. 7F) by suppression of SIRT3, a deacetylase participating in the regulation of CPT2 acetylation [18, 19], using 3-TYP (a SIRT3 inhibitor) obviously rescued the downregulation of CPT2 caused by SLC25A42 knockdown. Inversely, reduction of CPT2 acetylation (Fig. 7F) by activation of SIRT3 using Compound 5 v (a SIRT3 activator) markedly abolished the upregulation of CPT2 caused by forced SLC25A42 expression (Fig. 7F, G). These data suggest that SLC25A42 reprograms lipid metabolism in GC cells mainly by upregulating the acetylation and thus the expression of CPT2 in GC cells. Moreover, we found that the expressions of SLC25A42 and CPT2 were positively associated in tumor tissues from GC patients (Fig. 7H).

We next asked whether SLC25A42 inhibits ferroptosis by upregulating CPT2. The results in Fig. S4A–4C showed that overexpression of CPT2 obviously attenuated the promotion of ferroptosis caused by SLC25A42 silencing in MKN-1 cells, while knockdown of CPT2 rescued the suppression of ferroptosis caused by forced SLC25A42 expression in SNU-638 cells, suggesting that CPT2 are required for the suppression of ferroptosis by SLC25A42 in GC cells.

To further identify the acetyltransferase responsible for CPT2, the most common cell metabolism-related acetyltransferases (p300, PCAF, GCN5, TIP60, and HDAC6) [20] were over-expressed in GC cells. The results showed a clearly increase of CPT2 acetylation upon TIP60 overexpression (Fig S5A). Additionally, co-immunoprecipitation analysis showed a strong association between CPT2 and acetyltransferase TIP60 in GC cells (Fig. S5B), suggesting that TIP60 should be responsible for CPT2 acetylation in GC cells. To further confirm the acetylation site on CPT2, the two reported acetylation sites of CPT2 at K239 [21] and K79 [19] were mutated to arginines (R, nonacetylatable) via site-directed mutagenesis. The plasmids expressing CPT2-WT or two CPT2 mutants were then transfected into GC cells. The results showed that the acetylation level of CPT2-K79R increased significantly upon SLC25A42 overexpressing in GC cells, while no significant change in CPT2-K239R acetylation was observed when SLC25A42 was overexpressed (Fig. S5C), suggesting that SLC25A42 may promote the acetylation of CPT2 at K239 site.

## Discussion

The malignant characteristics of tumor cells, such as rapid proliferation and distant metastases, are intrinsically linked to their reprogrammed metabolism [22]. Mitochondria are important bioenergetics and biosynthetic organelles in eukaryotic cells. Accumulating evidence has suggested the relationship between mitochondrial dysfunction and cancer [6, 23]. However, key factors contributing to mitochondrial dysfunction and thus the initiation and progression gastric cancer remain not fully elucidated. Here, we demonstrate that SLC25A42 is upregulated and correlated with a worse prognosis in GC patients. SLC25A42 reprogrammed lipid metabolism in GC cells by upregulating the acetylation and thus the expression of CPT2 to promote cell survival and to suppresses ferroptosis.

The clinical data analysis revealed that SLC25A42 is upregulated and correlated with a worse prognosis in GC patients, implying a contribution of SLC25A42 to the progression of GC. However, the biological functions of SLC25A42 in the human malignancies have remained unexplored previously. Our in vitro and in vivo studies revealed that SLC25A42 promotes the proliferation while inhibiting cell death in GC cells, indicating a pivotal oncogenic role of SLC25A42 in GC. Moreover, using specific inhibitors targeting different types of programmed cell death, we found that SLC25A42 knockdown-induced GC cell death was notably rescued by suppression of ferroptosis with the Fer-1 treatment, suggesting that SLC25A42 suppresses ferroptosis in GC cells.

As a member of the solute carrier family 25 (SLC25), SLC25A42 has been reported to mediate the transport of coenzyme A (CoA) in mitochondria [8, 9]. However, the influence of SLC25A42 on cellular metabolism remains largely unknown, especially in human cancer cells. Besides glucose, fatty acid is another crucial source of energy in human cells through the oxidation in mitochondria. Upregulation of FAO has been reported to promote tumor cell proliferation, stemness, survival, and chemoresistance [24–28]. Although the abnormal fatty acid oxidation (FAO) has been reported in various cancer types, the link between mitochondrial dysfunction and the upregulation of FAO in cancer cells remains poorly understood. In this study, we revealed that SLC25A42 plays a crucial role in lipid metabolism reprogramming via acetylation-mediated stabilization of CPT2 and thus activation of FAO in GC cells. However, unlike our findings in GC cells, it was also found in platelet cells that CPT2 acetylation at K79 site blocked fatty acid oxidation. This contradiction may be explained by the fact that acetylation at different sites of CPT2 may have distinct impact on the activity and stability of CPT2, which requires further confirmation. In addition, we found that activation of FAO by SLC25A42 in GC cells suppressed ferroptosis of GC cells mainly by decreasing the levels of reactive oxidative species (ROS) and free fatty acids. Our data highlights SLC25A42 as a crucial suppressor of ferroptosis by activating FAO in cancer cells. Given that ferroptosis evasion has been reported as a critical mechanism of the resistance sorafenib [29], a tyrosine kinase inhibitor, and chemotherapy [30] in gastric cancer (GC), we postulate that silencing SLC25A42 might sensitize GC cells to ferroptotic death and enhances the anti-cancer activity of sorafenib and chemotherapy in GC, which still need more investigation.

In summary, our study revealed that upregulation of SLC25A42 promotes the growth while suppresses ferroptosis of GC cells by reprogramming lipid metabolism via upregulating the acetylation and thus expression of CPT2, suggesting that SLC25A42 represents a promising target for the treatment of GC.

## Supplementary Information

Supplementary Figures and Tables

Full and uncropped western blots

## Data Availability

The data supporting the findings of this study are available from the corresponding author upon reasonable request.

## References

[1] Smyth EC, Nilsson M, Grabsch HI, van Grieken NC, Lordick F. Gastric cancer. Lancet. 2020;396:635–48. 32861308 10.1016/S0140-6736(20)31288-5

[2] Zeng Y, Jin RU. Molecular pathogenesis, targeted therapies, and future perspectives for gastric cancer. Semin Cancer Biol. 2022;86:566–82. 34933124 10.1016/j.semcancer.2021.12.004 PMC12833737

[3] Monzel AS, Enriquez JA, Picard M. Multifaceted mitochondria: moving mitochondrial science beyond function and dysfunction. Nat Metab. 2023;5:546–62. 37100996 10.1038/s42255-023-00783-1 PMC10427836

[4] Liu Y, Sun Y, Guo Y, Shi X, Chen X, Feng W, et al. An overview: the diversified role of mitochondria in cancer metabolism. Int J Biol Sci. 2023;19:897–915. 36778129 10.7150/ijbs.81609 PMC9910000

[5] Genovese I, Carinci M, Modesti L, Aguiari G, Pinton P, Giorgi C. Mitochondria: insights into crucial features to overcome cancer chemoresistance. Int J Mol Sci. 2021;22:4770. 10.3390/ijms22094770 PMC8124268 33946271

[6] Vasan K, Werner M, Chandel NS. Mitochondrial metabolism as a target for cancer therapy. Cell Metab. 2020;32:341–52. 32668195 10.1016/j.cmet.2020.06.019 PMC7483781

[7] Rochette L, Meloux A, Zeller M, Malka G, Cottin Y, Vergely C. Mitochondrial SLC25 carriers: novel targets for cancer therapy. Molecules 2020;25:2417. 10.3390/molecules25102417 PMC7288124 32455902

[8] Fiermonte G, Paradies E, Todisco S, Marobbio CM, Palmieri F. A novel member of solute carrier family 25 (SLC25A42) is a transporter of coenzyme A and adenosine 3’,5’-diphosphate in human mitochondria. J Biol Chem. 2009;284:18152–9. 19429682 10.1074/jbc.M109.014118 PMC2709381

[9] Iuso A, Alhaddad B, Weigel C, Kotzaeridou U, Mastantuono E, Schwarzmayr T, et al. A homozygous splice site mutation in SLC25A42, encoding the mitochondrial transporter of coenzyme a, causes metabolic crises and epileptic encephalopathy. JIMD Rep. 2019;44:1–7. 29923093 10.1007/8904_2018_115 PMC6323019

[10] Barritt SA, DuBois-Coyne SE, Dibble CC. Coenzyme A biosynthesis: mechanisms of regulation, function and disease. Nat Metab. 2024;6:1008–23. 38871981 10.1038/s42255-024-01059-y

[11] Shamseldin HE, Smith LL, Kentab A, Alkhalidi H, Summers B, Alsedairy H, et al. Mutation of the mitochondrial carrier SLC25A42 causes a novel form of mitochondrial myopathy in humans. Hum Genet. 2016;135:21–30. 26541337 10.1007/s00439-015-1608-8 PMC4900140

[12] Aldosary M, Baselm S, Abdulrahim M, Almass R, Alsagob M, AlMasseri Z, et al. SLC25A42-associated mitochondrial encephalomyopathy: report of additional founder cases and functional characterization of a novel deletion. JIMD Rep. 2021;60:75–87. 34258143 10.1002/jmd2.12218 PMC8260478

[13] Gyorffy B. Integrated analysis of public datasets for the discovery and validation of survival-associated genes in solid tumors. Innovation. 2024;5:100625. 38706955 10.1016/j.xinn.2024.100625 PMC11066458

[14] Rath S, Sharma R, Gupta R, Ast T, Chan C, Durham TJ, et al. MitoCarta3.0: an updated mitochondrial proteome now with sub-organelle localization and pathway annotations. Nucleic Acids Res. 2021;49:D1541–D1547. 33174596 10.1093/nar/gkaa1011 PMC7778944

[15] Trefely S, Lovell CD, Snyder NW, Wellen KE. Compartmentalised acyl-CoA metabolism and roles in chromatin regulation. Mol Metab. 2020;38:100941. 32199817 10.1016/j.molmet.2020.01.005 PMC7300382

[16] He W, Li Q, Li X. Acetyl-CoA regulates lipid metabolism and histone acetylation modification in cancer. Biochim Biophys Acta Rev Cancer. 2023;1878:188837. 36403921 10.1016/j.bbcan.2022.188837

[17] Wu Z, Guan KL. Acetyl-CoA, protein acetylation, and liver cancer. Mol Cell. 2022;82:4196–8. 36400006 10.1016/j.molcel.2022.10.015

[18] Junli Z, Shuhan W, Yajuan Z, Xiaoling D, Jiahuan L, Keshu X. The role and mechanism of CREBH regulating SIRT3 in metabolic associated fatty liver disease. Life Sci. 2022;306:120838. 35902030 10.1016/j.lfs.2022.120838

[19] Fan X, Wang Y, Cai X, Shen Y, Xu T, Xu Y, et al. CPT2 K79 acetylation regulates platelet life span. Blood Adv. 2022;6:4924–35. 35728063 10.1182/bloodadvances.2021006687 PMC9631617

[20] You Z, Jiang WX, Qin LY, Gong Z, Wan W, Li J, et al. Requirement for p62 acetylation in the aggregation of ubiquitylated proteins under nutrient stress. Nat Commun. 2019;10:5792. 31857589 10.1038/s41467-019-13718-w PMC6923396

[21] Guo Y, Zhang Z, Wen Z, Kang X, Wang D, Zhang L, et al. Mitochondrial SIRT2-mediated CPT2 deacetylation prevents diabetic cardiomyopathy by impeding cardiac fatty acid oxidation. Int J Biol Sci. 2025;21:725–44. 39781464 10.7150/ijbs.102834 PMC11705638

[22] Tufail M, Jiang CH, Li N. Altered metabolism in cancer: insights into energy pathways and therapeutic targets. Mol Cancer. 2024;23:203. 39294640 10.1186/s12943-024-02119-3 PMC11409553

[23] Missiroli S, Perrone M, Genovese I, Pinton P, Giorgi C. Cancer metabolism and mitochondria: finding novel mechanisms to fight tumours. Ebiomedicine. 2020;59:102943. 32818805 10.1016/j.ebiom.2020.102943 PMC7452656

[24] Ma Y, Temkin SM, Hawkridge AM, Guo C, Wang W, Wang XY, et al. Fatty acid oxidation: an emerging facet of metabolic transformation in cancer. Cancer Lett. 2018;435:92–100. 30102953 10.1016/j.canlet.2018.08.006 PMC6240910

[25] Zhou L, Luo Y, Liu Y, Zeng Y, Tong J, Li M, et al. Fatty acid oxidation mediated by malonyl-CoA decarboxylase represses renal cell carcinoma progression. Cancer Res. 2023;83:3920–39. 37729394 10.1158/0008-5472.CAN-23-0969 PMC10690093

[26] Li H, Song J, He Y, Liu Y, Liu Z, Sun W, et al. CRISPR/Cas9 screens reveal that hexokinase 2 enhances cancer stemness and tumorigenicity by activating the ACSL4-fatty acid beta-oxidation pathway. Adv Sci. 2022;9:e2105126. 10.1002/advs.202105126 PMC9313492 35603967

[27] Li J, Xia Q, Di C, Li C, Si H, Zhou B, et al. Tumor cell-intrinsic CD96 mediates chemoresistance and cancer stemness by regulating mitochondrial fatty acid beta-oxidation. Adv Sci. 2023;10:e2202956. 10.1002/advs.202202956 PMC9982582 36581470

[28] Huang Y, Wang F, Lin X, Li Q, Lu Y, Zhang J, et al. Nuclear VCP drives colorectal cancer progression by promoting fatty acid oxidation. Proc Natl Acad Sci USA. 2023;120:e2221653120. 37788309 10.1073/pnas.2221653120 PMC10576098

[29] Xu X, Li Y, Wu Y, Wang M, Lu Y, Fang Z, et al. Increased ATF2 expression predicts poor prognosis and inhibits sorafenib-induced ferroptosis in gastric cancer. Redox Biol. 2023;59:102564. 36473315 10.1016/j.redox.2022.102564 PMC9723522

[30] Ouyang S, Li H, Lou L, Huang Q, Zhang Z, Mo J, et al. Inhibition of STAT3-ferroptosis negative regulatory axis suppresses tumor growth and alleviates chemoresistance in gastric cancer. Redox Biol. 2022;52:102317. 35483272 10.1016/j.redox.2022.102317 PMC9108091

