# Supplementary material for: SLC25A42 promotes gastric cancer growth by conferring ferroptosis resistance through enhancing CPT2-mediated fatty acid oxidation
Source: Cell Death Dis. 2025 Apr 17;16(1):309. doi: 10.1038/s41419-025-07644-7 (PMC12006318; doi:10.1038/s41419-025-07644-7)
Supplement: Supplementary file 1 — Supplementary Figures and Tables [file 41419_2025_7644_MOESM1_ESM.doc]

**Supplemental information**

**SLC25A42 promotes gastric cancer growth by conferring ferroptosis resistance through enhancing CPT2-mediated fatty acid oxidation**

**Supplemental figures**

**Figure S1.** **SLC25A42 promotes the proliferation of gastric cancer (GC) cells.** (A and B) The efficiencies of SLC25A42 knockdown and overexpression were tested by qRT-PCR (A) and Western blot (B) assays in indicated gastric cancer cells. (C) Flow cytometry was utilized to investigate the progression of cell cycle in GC cells. (D) EdU assay was undertaken for assessment of cell proliferation in GC cells. **P* < 0.05; ns, not significant.


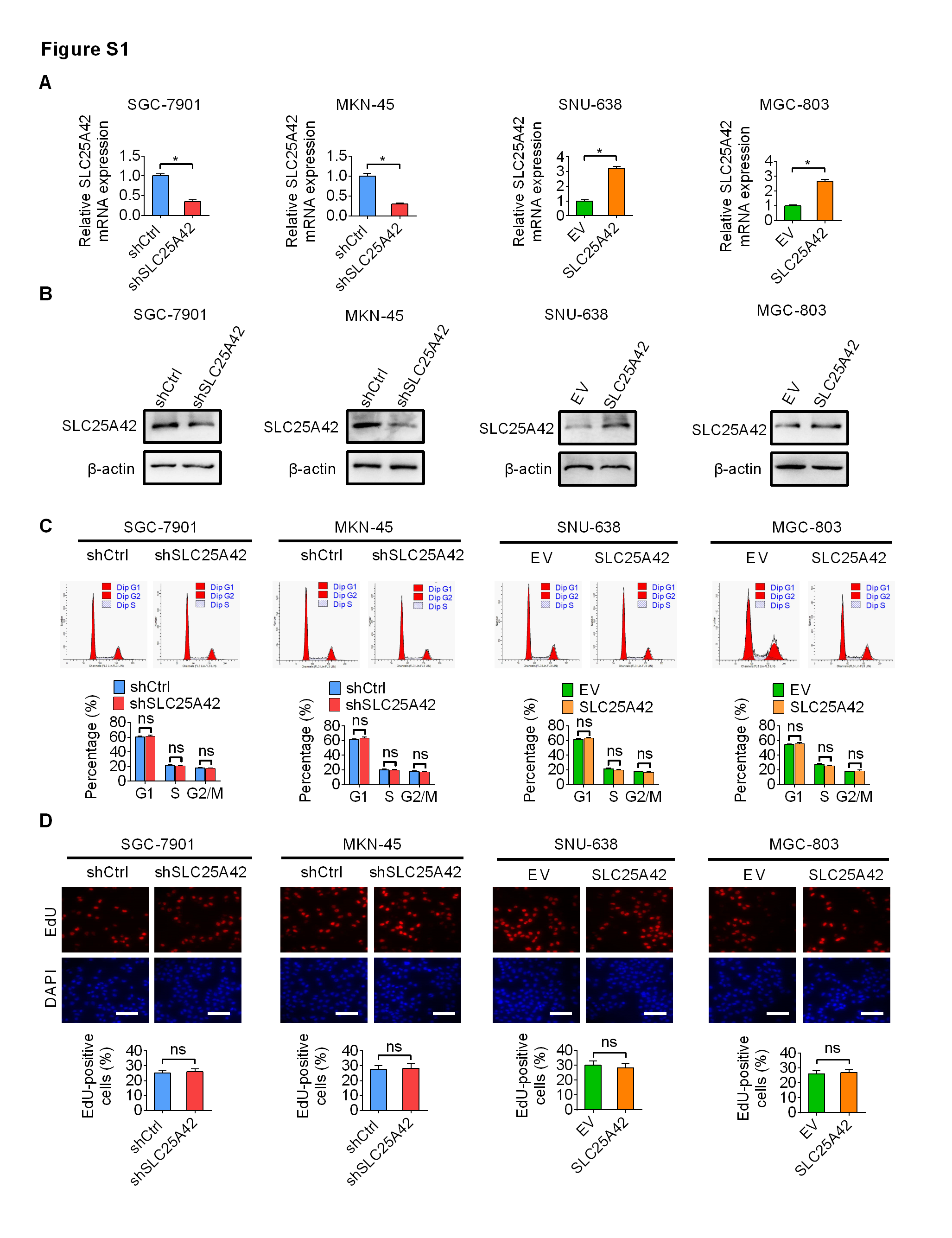


**Figure S2.** IHC staining of 4-hydroxy-2-noneal (4-HNE) in xenograft tumor tissues from nude mice injected with SLC25A42 knocking-down or corresponding control SGC-7901 cells (n=6). **P* < 0.05.

**
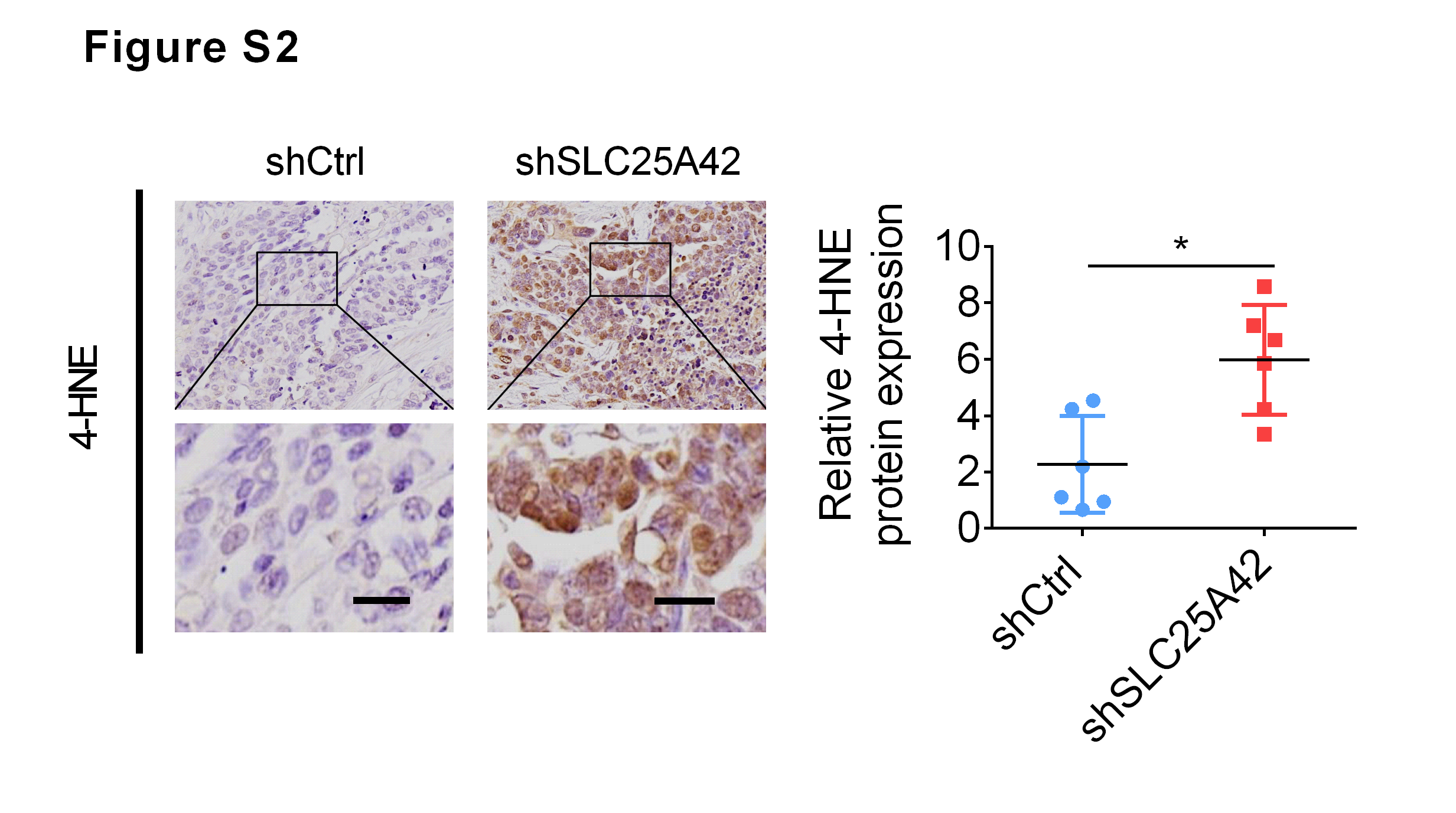
**

**Figure S3.** Double immunofluorescence staining of SLC25A42 and mitochondrial in SGC-7901 and MKN-45 cells.


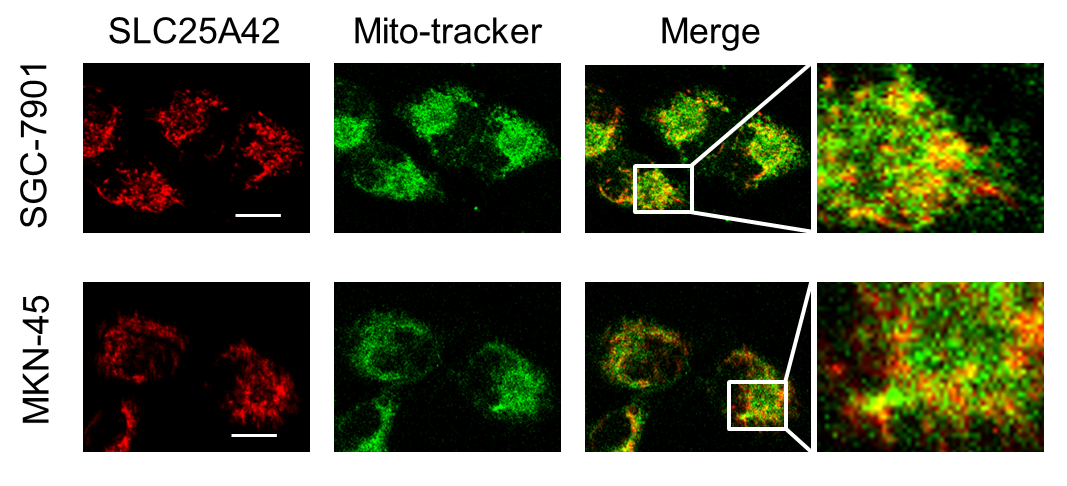


**Figure S4.** (A) Flow cytometry was utilized to investigate the rates of apoptosis in MKN-1 and SNU-638 cells with indicated treatment.. (B-C) The levels of lipid peroxidation (B) and intracellular Fe^2+^ (C) were assessed in MKN-1 and SNU-638 cells with indicated treatment. Scale bar, 10 μm. **P* < 0.05.

**
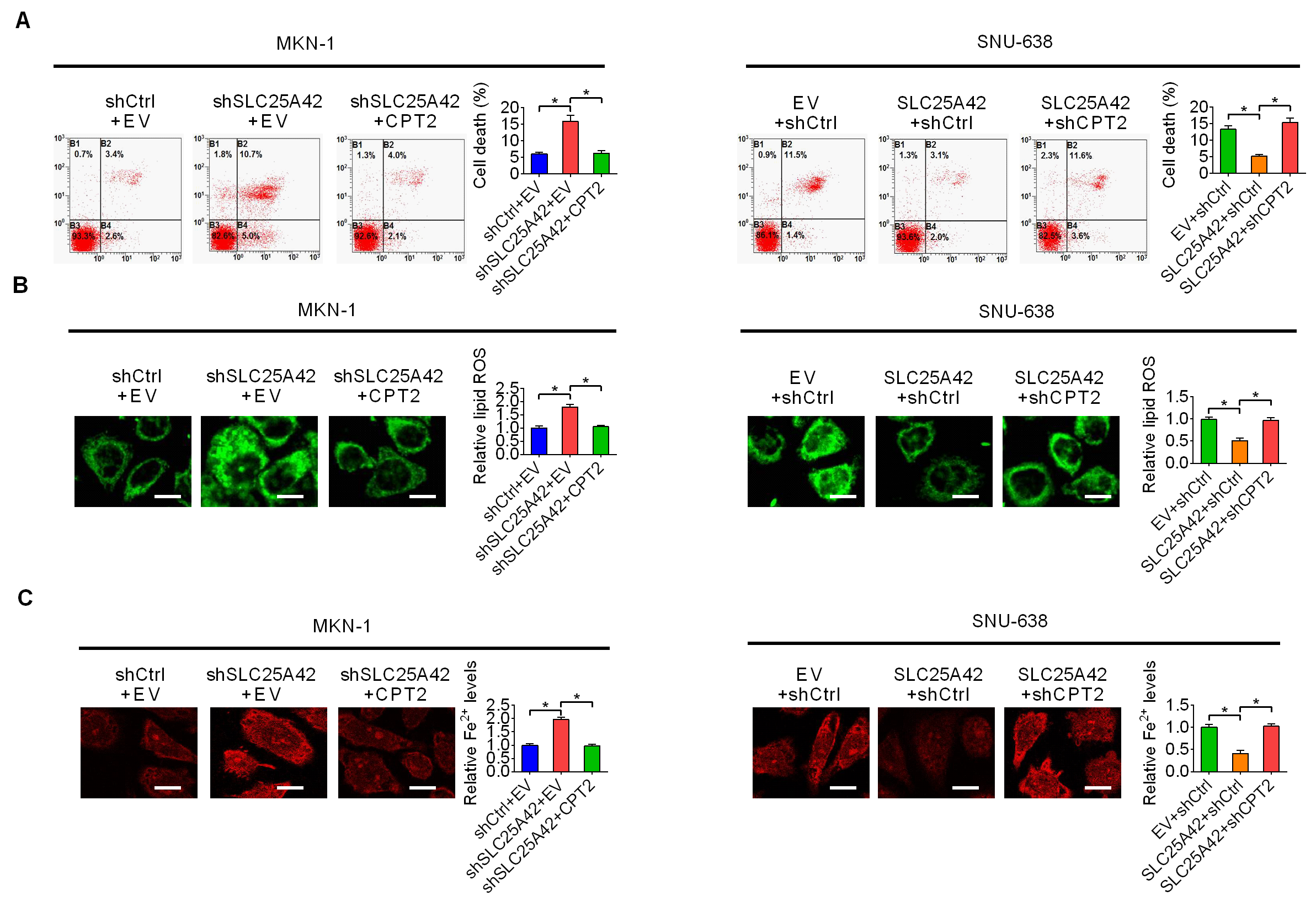
**

**Figure S5.** (A) The acetylation of CPT2 was assessed by western blot assay in SNU-638 cells. (B) Co-immunoprecipitation analysis for association between CPT2 and acetyltransferase TIP60 in SNU-638 cells. (C) SNU-638 cells with SLC25A42 overexpression were transfected with either CPT2-WT or mutant plasmids, including K79R and K239R.


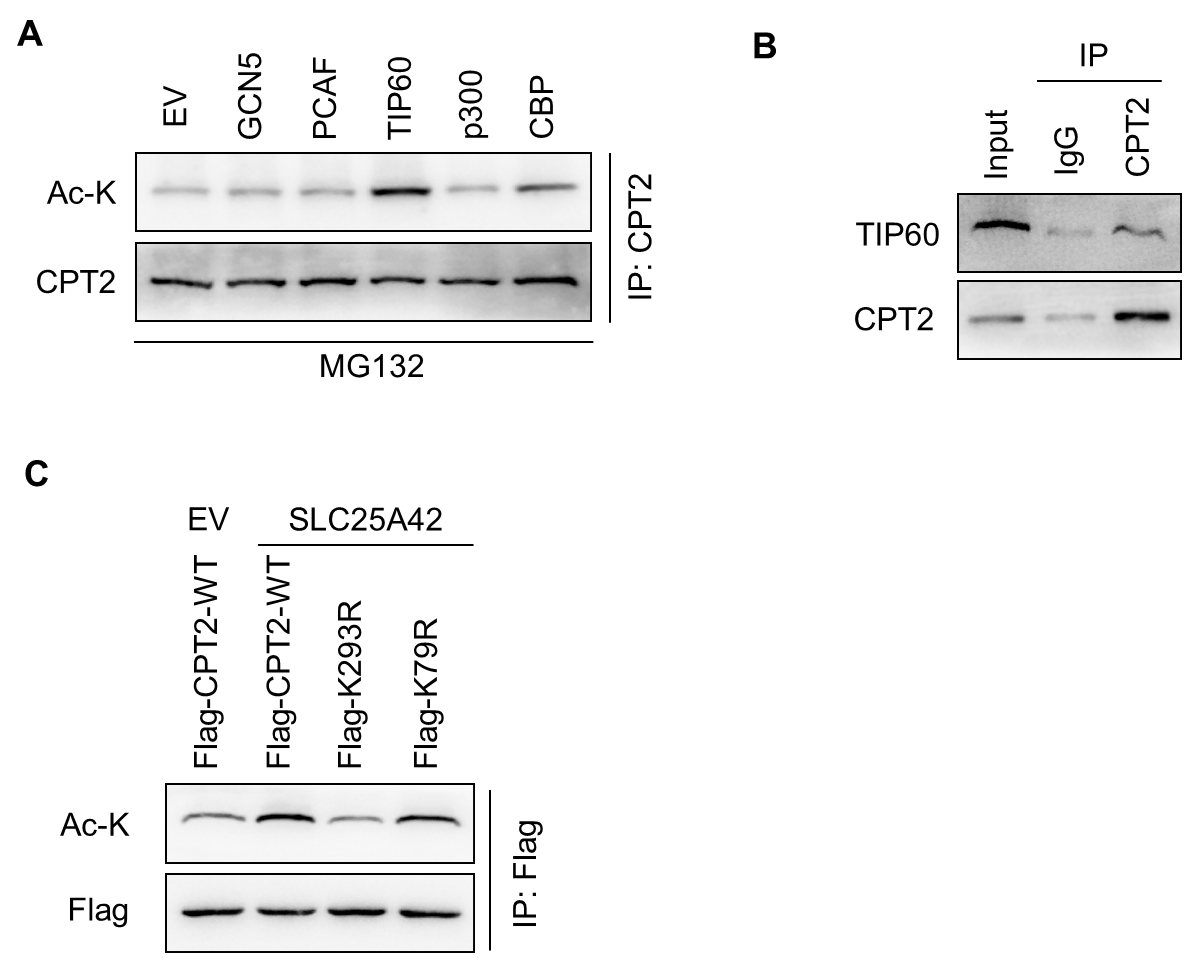


**Supplementary Tables**

**Table S1.** Primers used for PCR assay.

| **Gene** | **Forward Primer** | **Reverse Primer** |
| --- | --- | --- |
| *SLC25A42* | TGGTGCCCTTGCCAAAACA | CCCTCGTTGAGGTAGGTGTAG |
| *ACSL1* | CTTATGGGCTTCGGAGCTTTT | CAAGTAGTGCGGATCTTCGTG |
| *CPT1A* | TCCAGTTGGCTTATCGTGGTG | TCCAGAGTCCGATTGATTTTTGC |
| *CPT2* | CTGGAGCCAGAAGTGTTCCAC | AGGCACAAAGCGTATGAGTCT |
| *ACOX1* | ACTCGCAGCCAGCGTTATG | AGGGTCAGCGATGCCAAAC |
| *β-actin* | AGGCACCAGGGCGTGAT | GCCCACATAGGAATCCTTCTGAC |

**Table S2.** Primary antibodies used for IHC and Western blot assays

| **Antibody** | **Company (Cat. No.)** | **Working dilutions** |
| --- | --- | --- |
| SLC25A42 | NOVUS (NBP2-13325) | IHC, 1/400; WB, 1/2000 |
| Ki-67 | Proteintech (27309-1-AP) | IHC, 1/500 |
| ACSL1 | Proteintech (13989-1-AP) | WB, 1/1000 |
| CPT1A | Proteintech (15184-1-AP) | WB, 1/1000 |
| CPT2 | Abcam (ab181114) | WB, 1/1000; IHC, 1/300 |
| ACOX1 | Proteintech (10957-1-AP) | WB, 1/1000 |
| acetyl Lysine | Abcam (ab190479) | IHC, 1/400 |
| TIP60 | Proteintech (10827-1-A) | IP: 1/200 |
| β-actin | Proteintech (20536-1-AP) | WB: 1/1000 |

**Table S3. Correlation between the expressions of SLC25A42 and clinicopathologic features in 385 GC patients.**

| Variables | No. of cases (%) | SLC25A42 expression | | *P* value |
| --- | --- | --- | --- | --- |
|  |  | Low | High |  |
| All | 385 (100%) | 193 | 192 |  |
| Age |  |  |  |  |
| <65 | 184 (47.8%) | 95 | 89 | 0.610 |
| >=65 | 201 (52.2%) | 98 | 103 |  |
| Gender |  |  |  |  |
| Female | 142 (36.9%) | 68 | 74 | 0.527 |
| Male | 243 (63.1%) | 125 | 118 |  |
| Differentiation |  |  |  |  |
| High/Median | 175 (45.5%) | 84 | 91 | 0.474 |
| Low | 210 (54.5%) | 109 | 101 |  |
| Lauren’s classification |  |  |  |  |
| Intestinal | 136 (35.3%) | 63 | 73 | 0.287 |
| Diffuse/Mixed | 249 (64.7%) | 130 | 119 |  |
| Size |  |  |  |  |
| <5 | 237 (61.6%) | 131 | 106 | **0.012** |
| >=5 | 148 (38.4%) | 62 | 86 |  |
| Lymphatic invasion |  |  |  |  |
| No | 118 (30.6%) | 53 | 65 | 0.186 |
| Yes | 267 (69.4%) | 140 | 127 |  |
| Stage |  |  |  |  |
| I+ II | 183 (47.5%) | 102 | 81 | **0.041** |
| III+ IV | 202 (52.5%) | 91 | 111 |  |
