# Supplementary material for: SLC25A42 promotes gastric cancer growth by conferring ferroptosis resistance through enhancing CPT2-mediated fatty acid oxidation
Source: Cell Death Dis. 2025 Apr 17;16(1):309. doi: 10.1038/s41419-025-07644-7 (PMC12006318; doi:10.1038/s41419-025-07644-7)
Supplement: Supplementary file 2 — Full and uncropped western blots [file 41419_2025_7644_MOESM2_ESM.pdf]

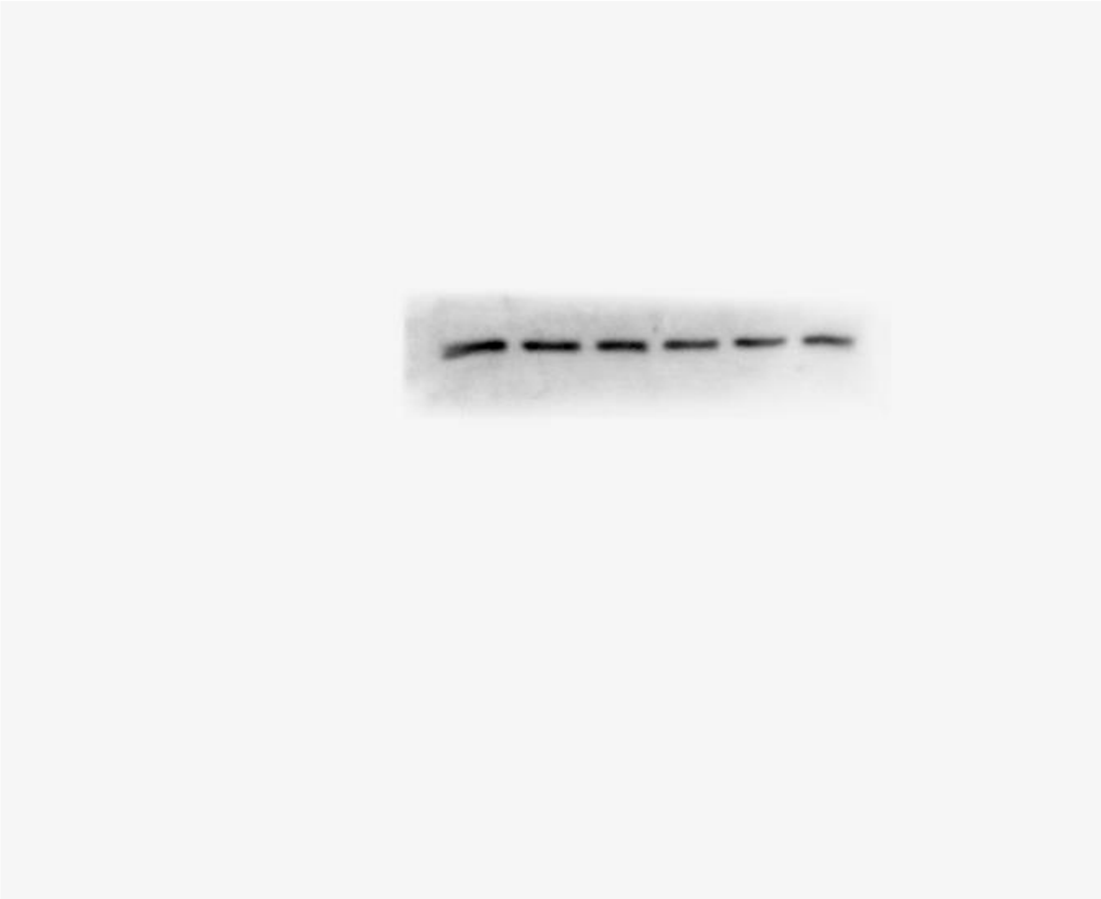

Fig 1D (IB: SLC25A44)

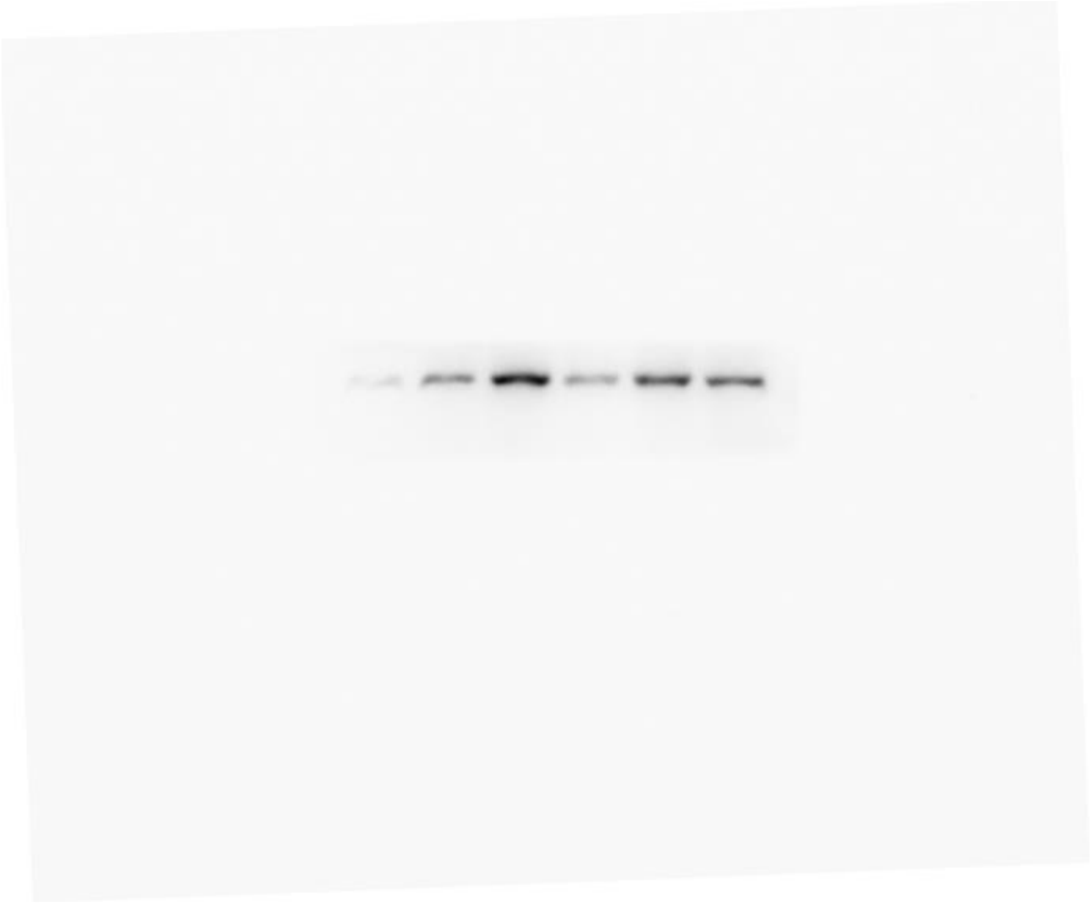

Fig 1D (IB: β-actin)

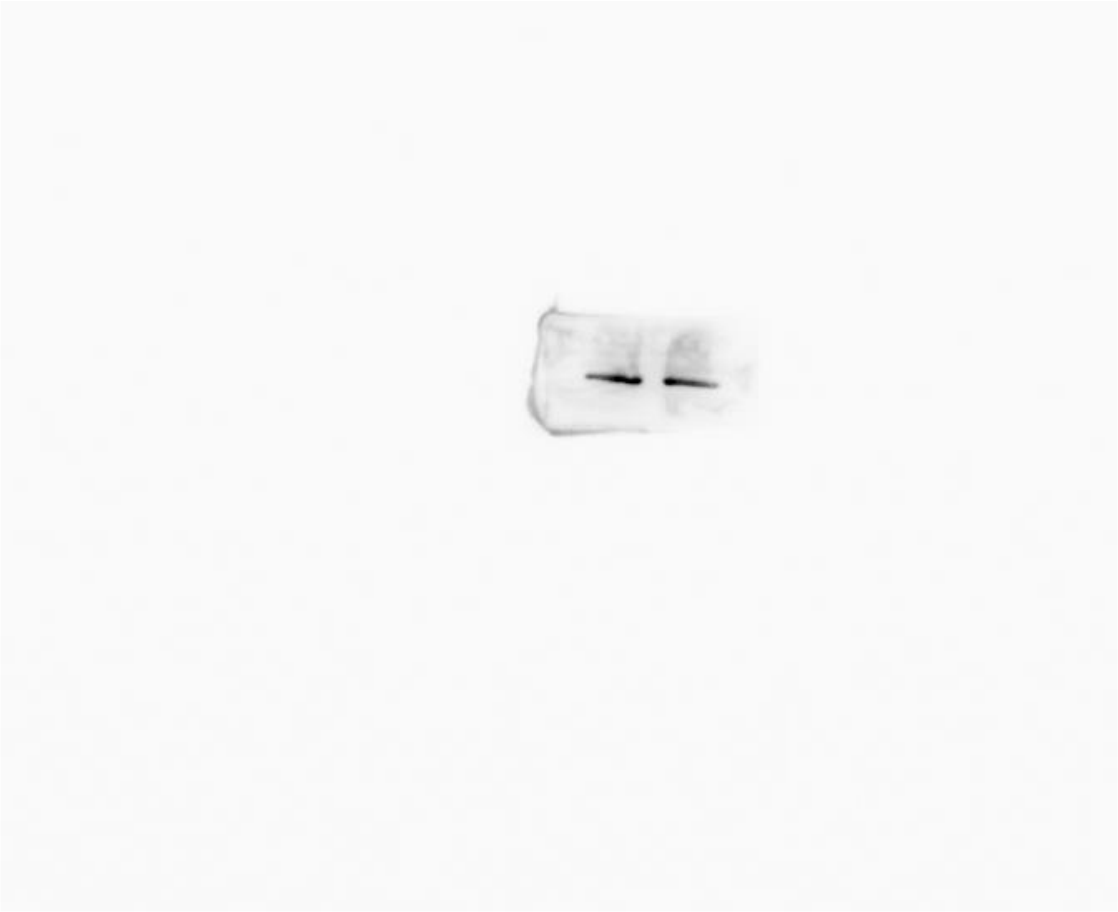

Fig 7B (IB: ACSL1 in MKN-1 cells)

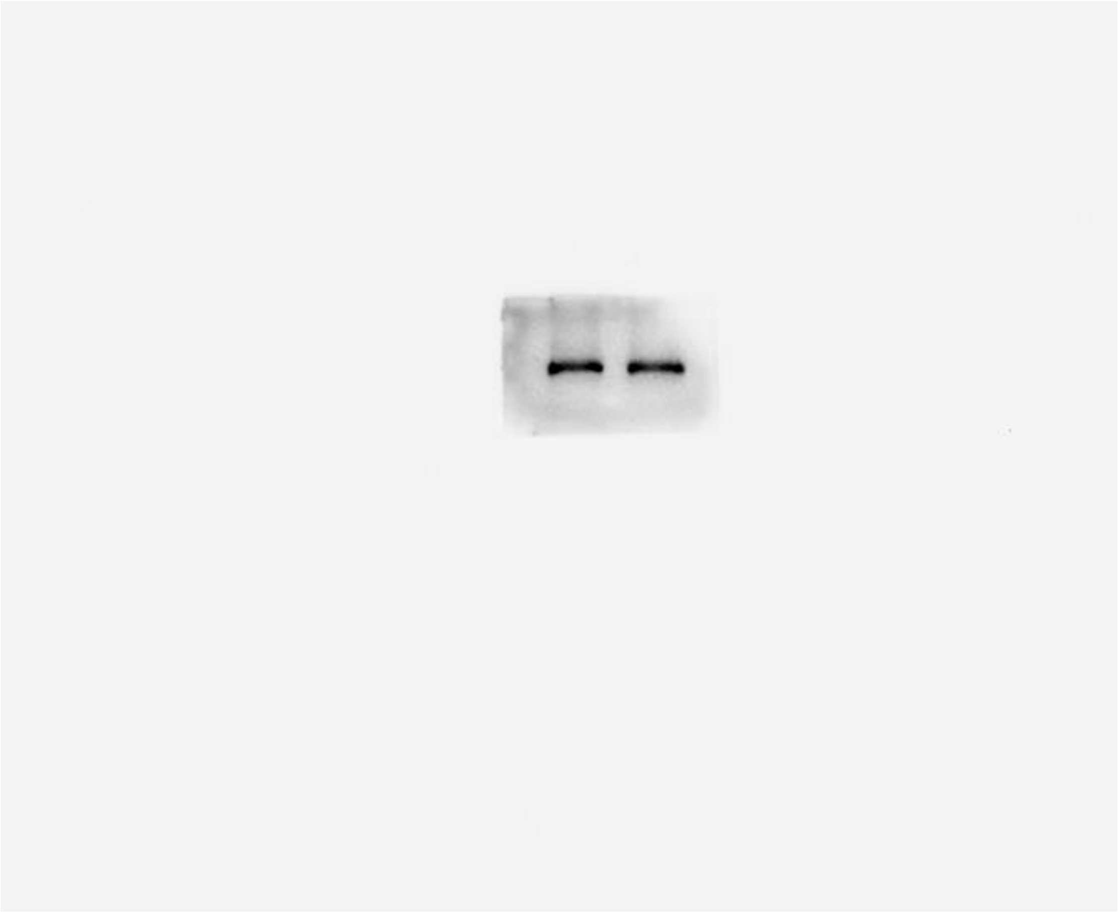

Fig 7B (IB: ACSL1 in SNU-638 cells)

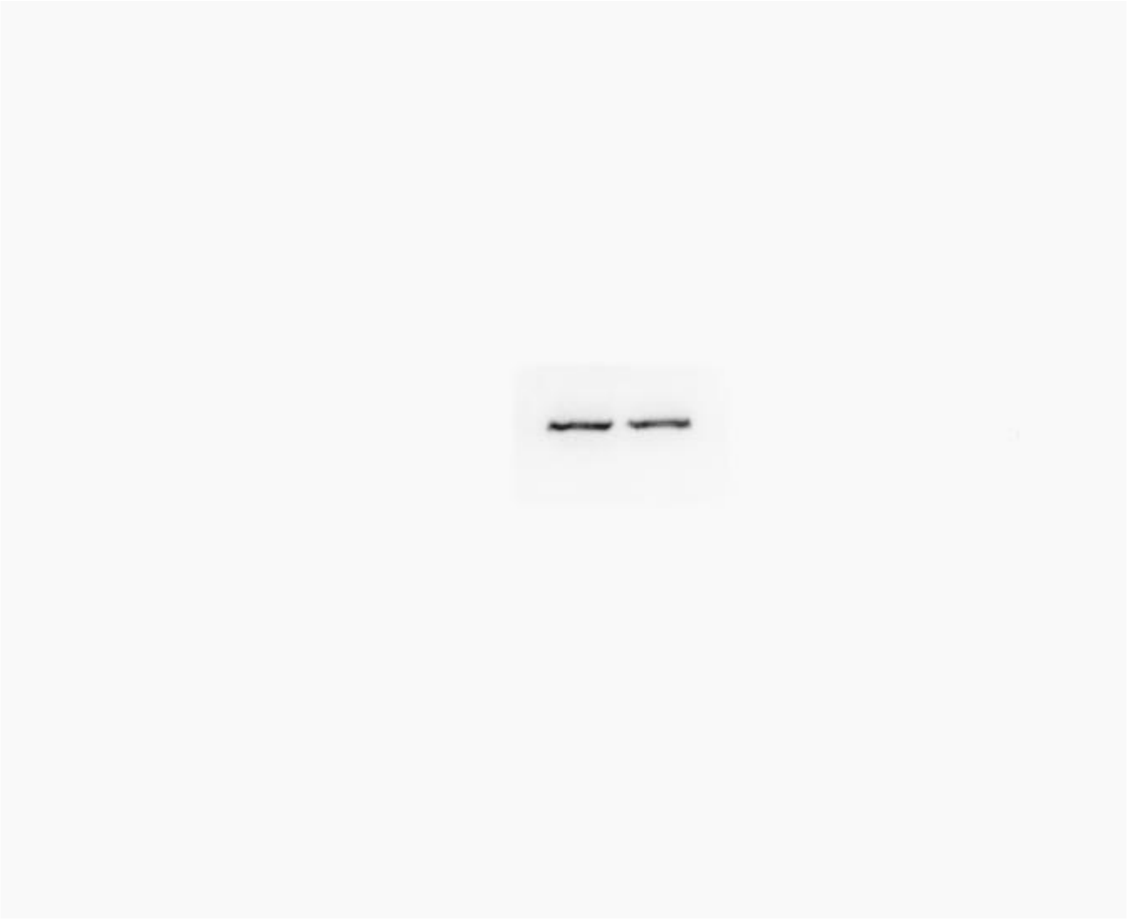

Fig 7B (IB: CPT1A in MKN-1 cells)

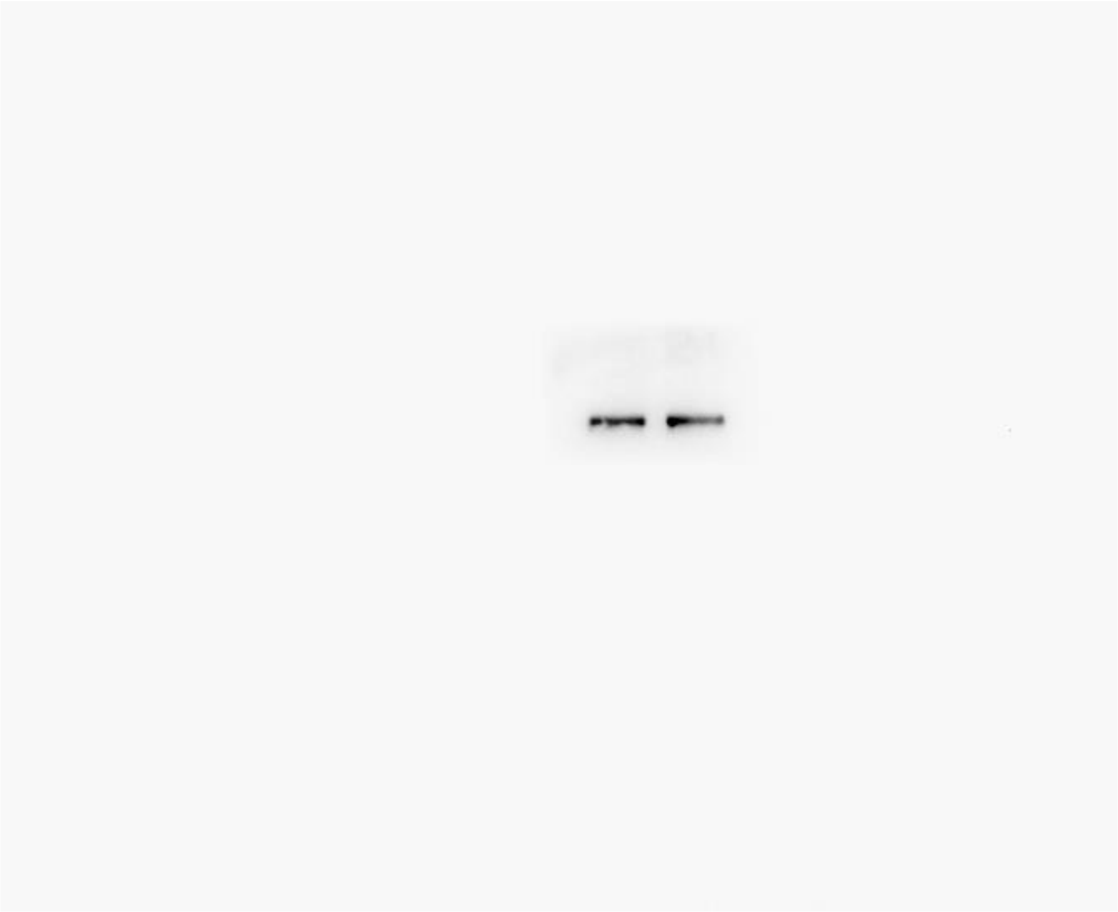

Fig 7B (IB: CPT1A in SNU-638 cells)

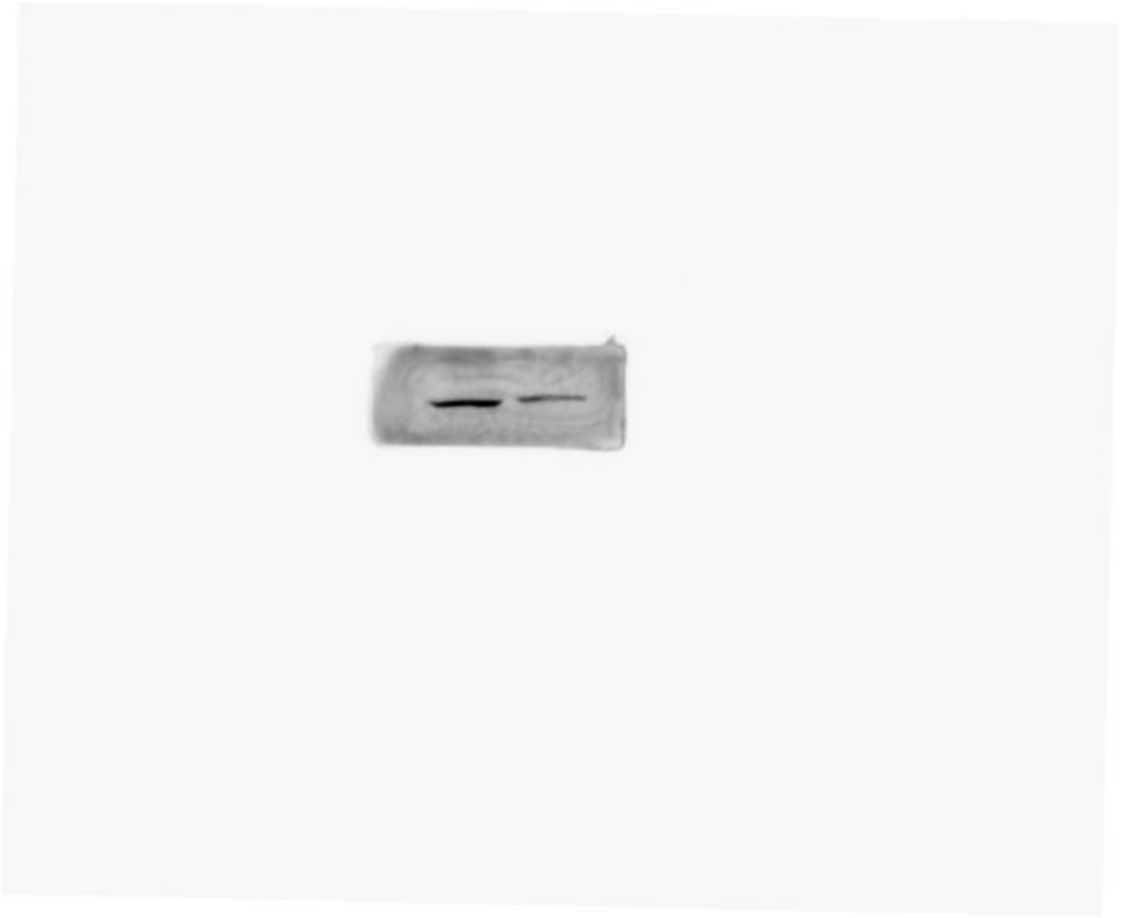

Fig 7B (IB: CPT2 in MKN-1 cells)

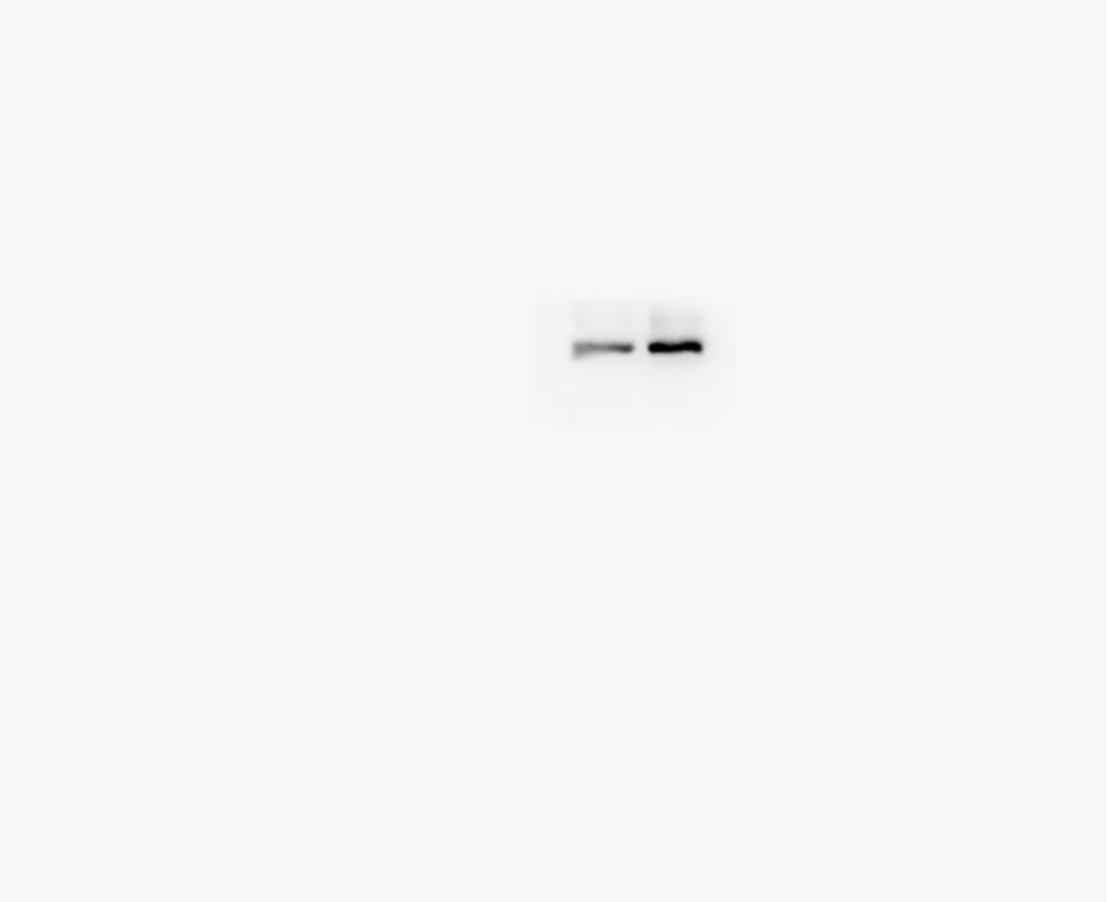

Fig 7B (IB: CPT2 in SNU-638 cells)

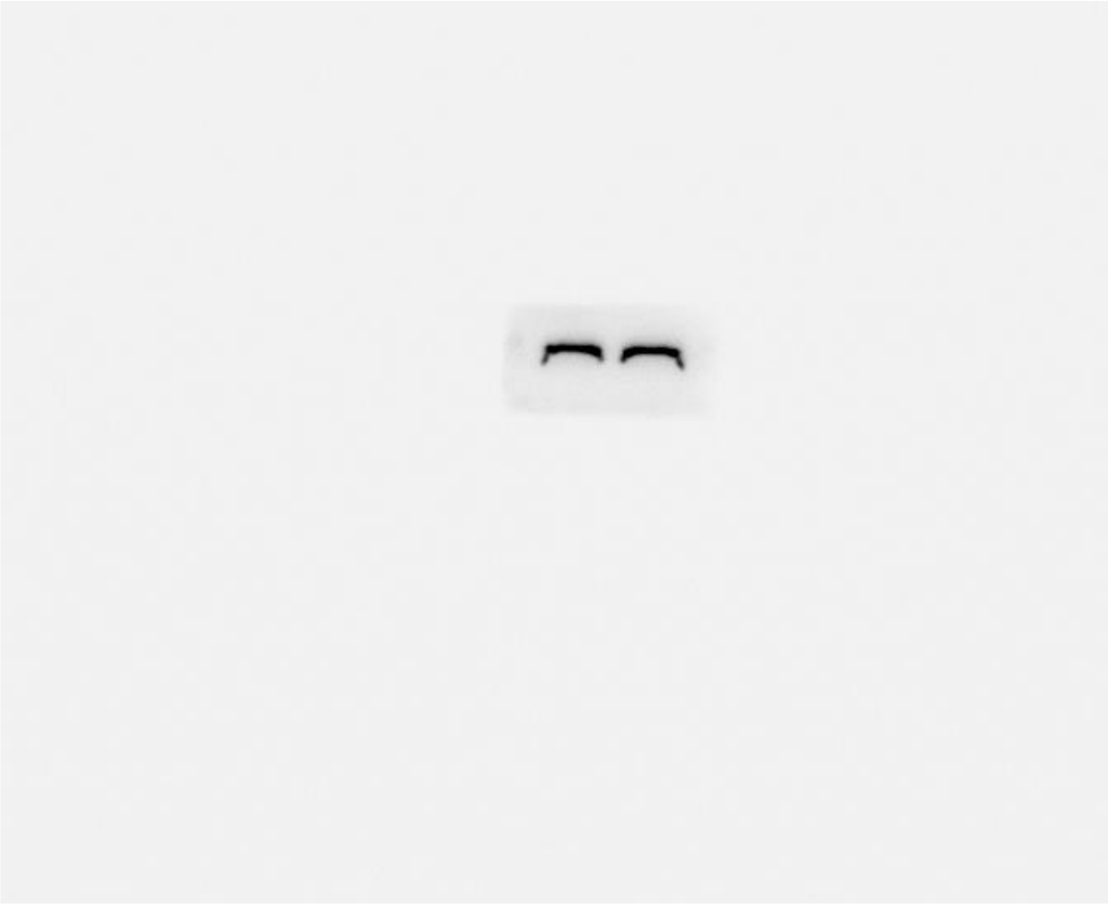

Fig 7B (IB: ACOX1 in MKN-1 cells)

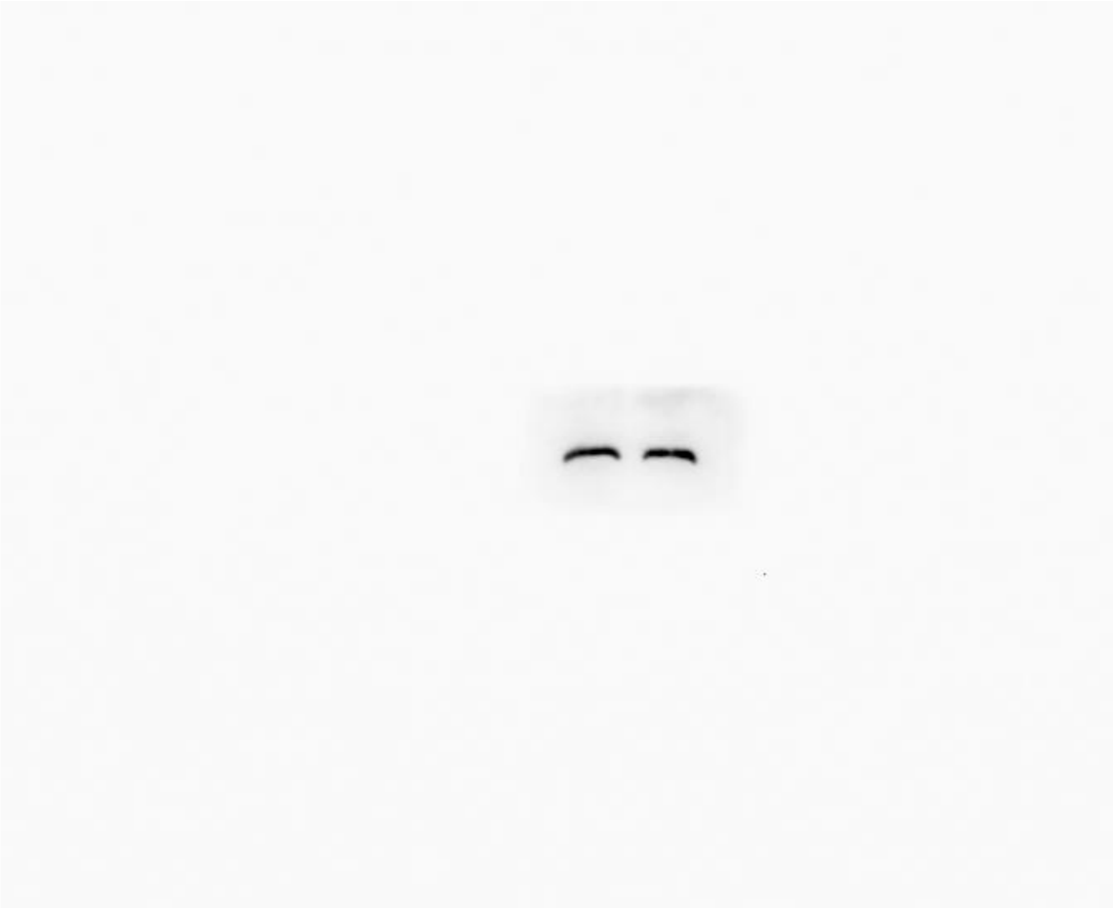

Fig 7B (IB: ACOX1 in SNU-638 cells)

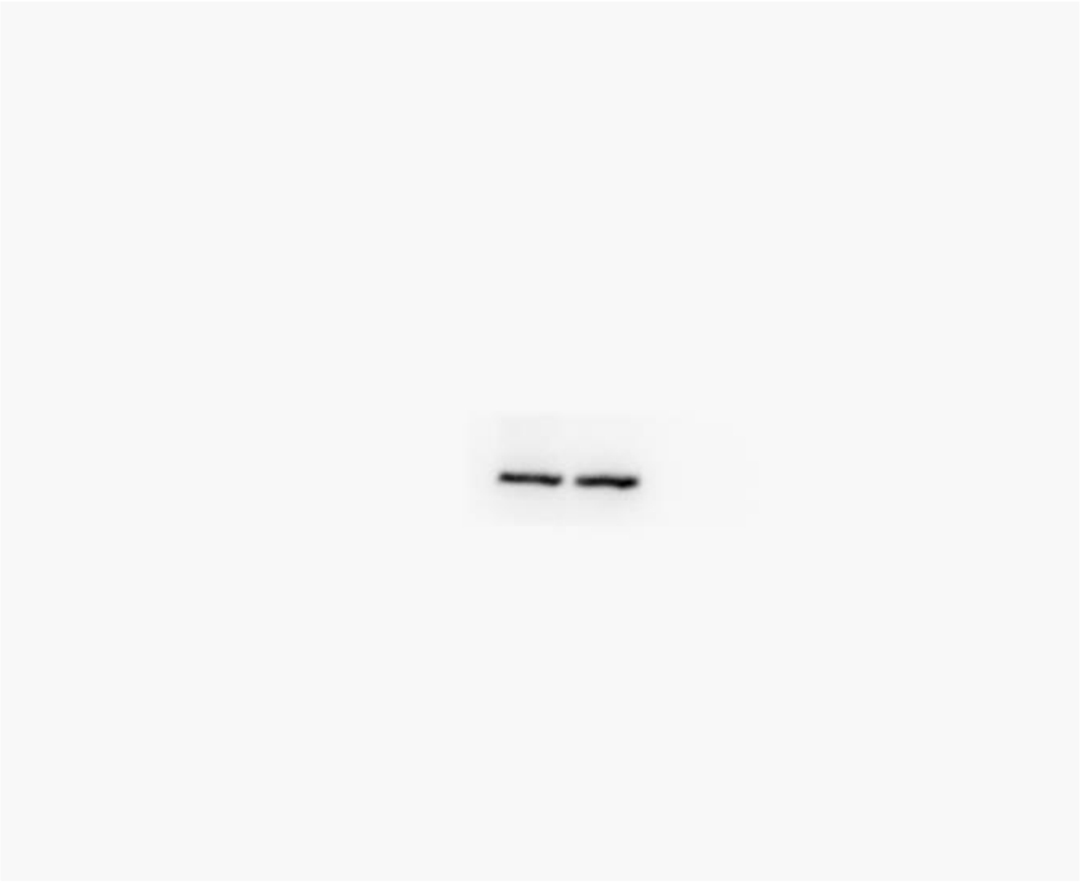

Fig 7B (IB: β-actin in MKN-1 cells)

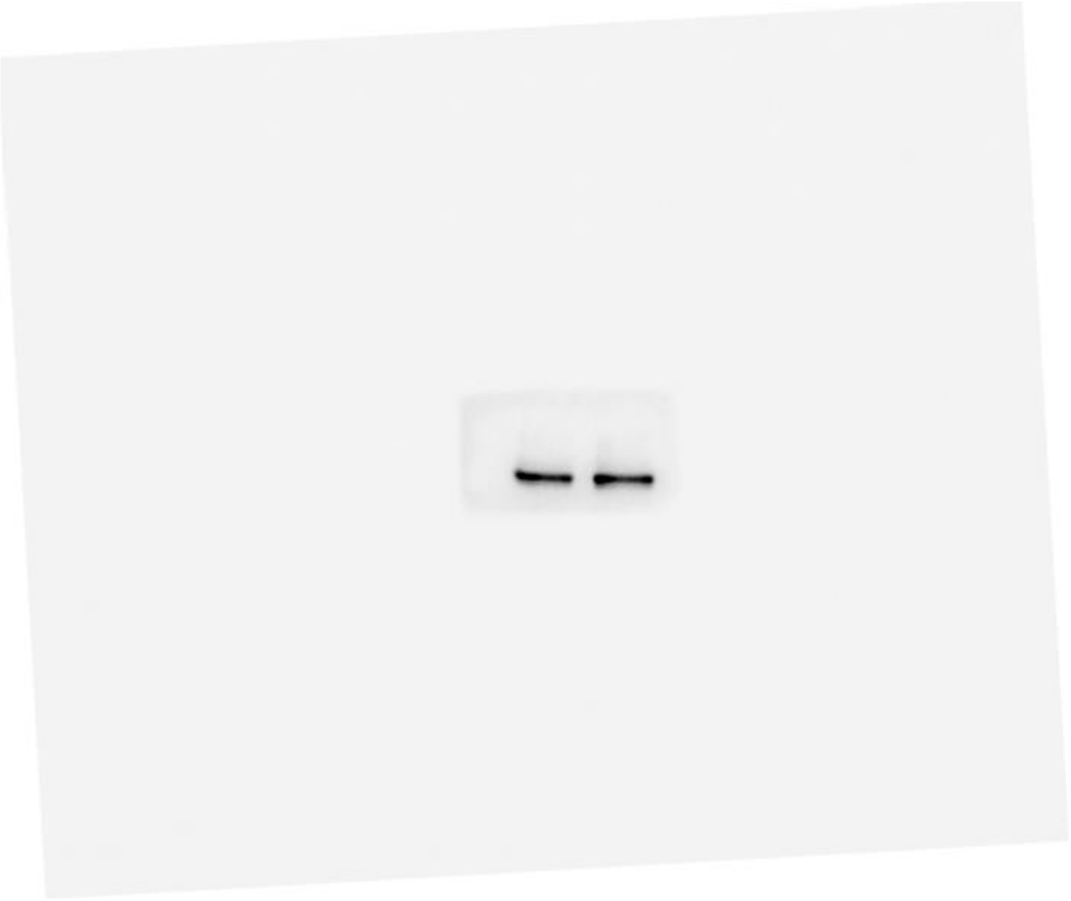

Fig 7B (IB: β-actin in SNU-638 cells)

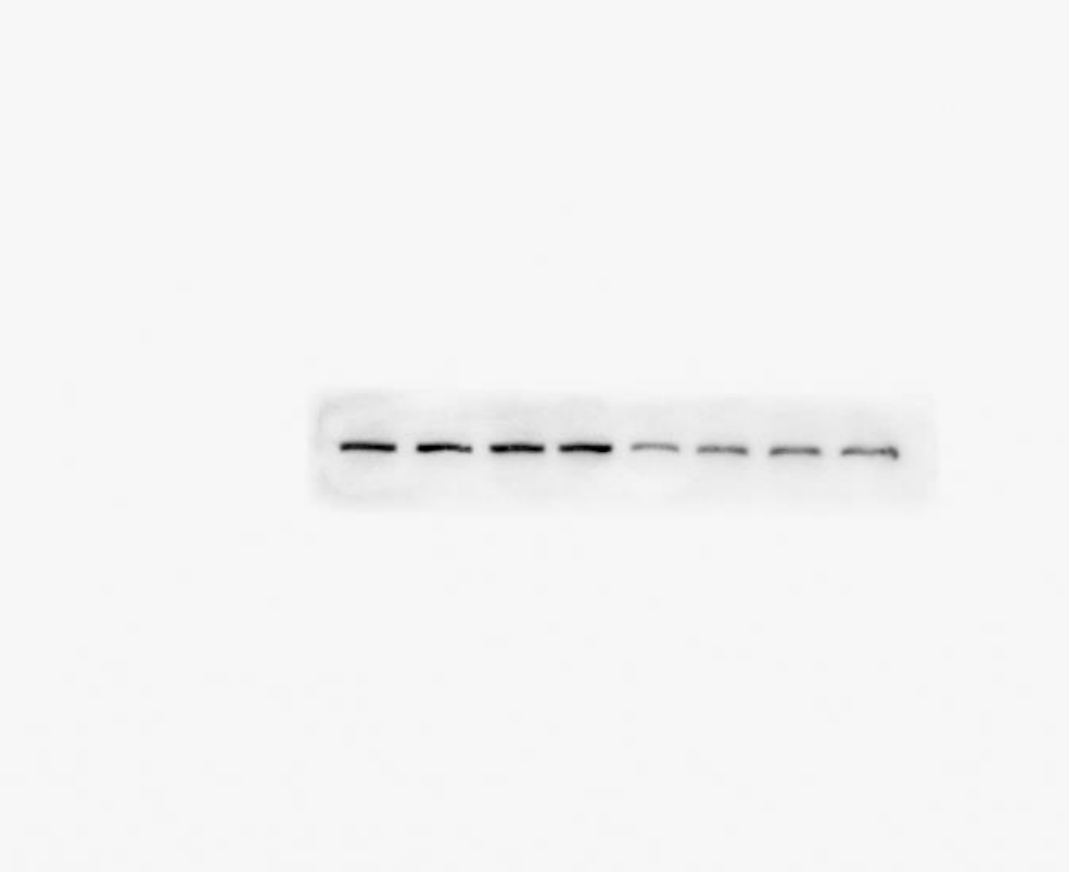

Fig 7C (IB: SLC25A42 in MKN-1 cells)

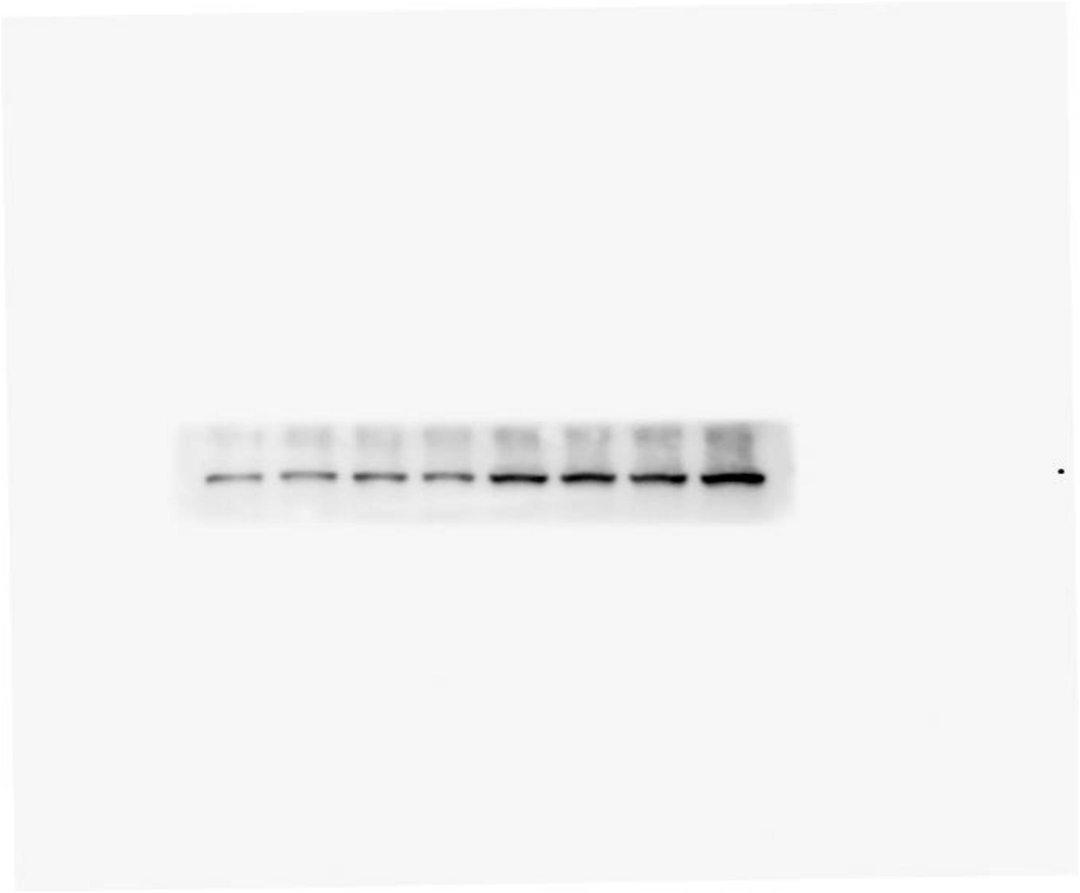

Fig 7C (IB: SLC25A42 in SNU-638 cells)

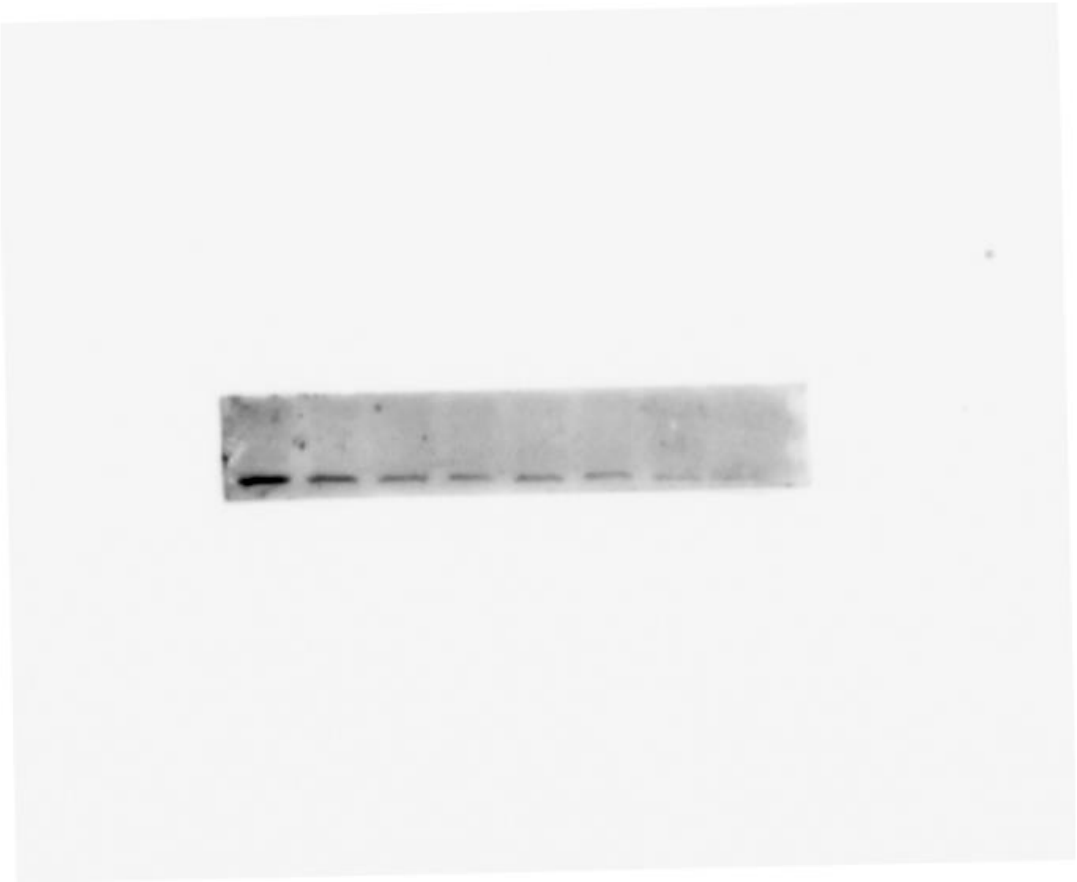

Fig 7C (IB: CPT2 in MKN-1 cells)

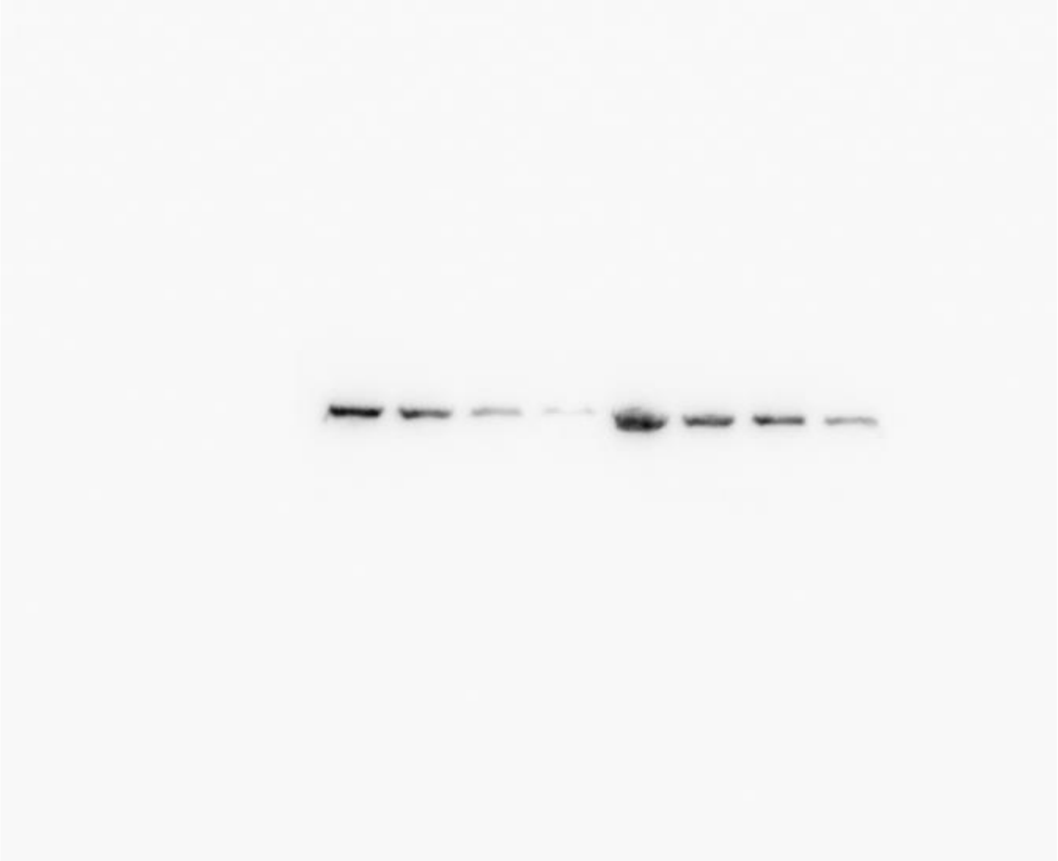

Fig 7C (IB: CPT2 in SNU-638 cells)

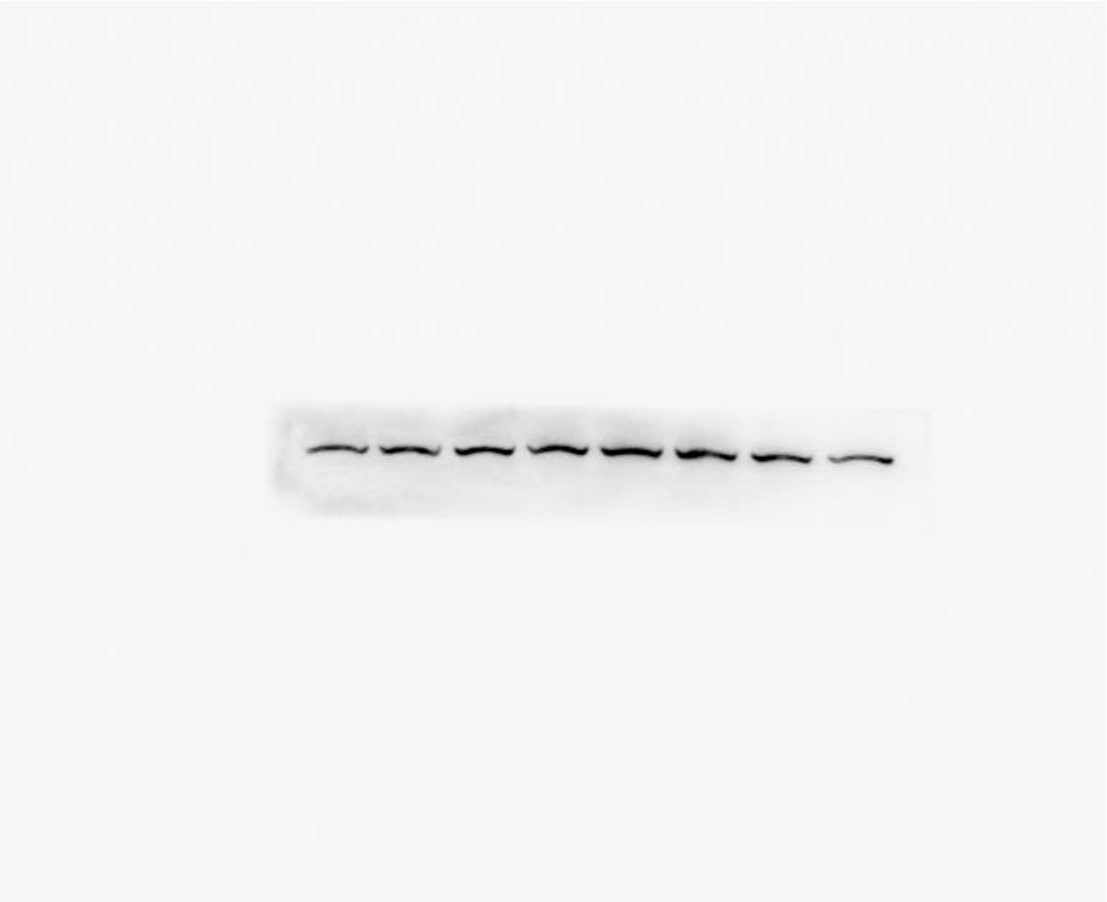

Fig 7C (IB: β-actin in MKN-1 cells)

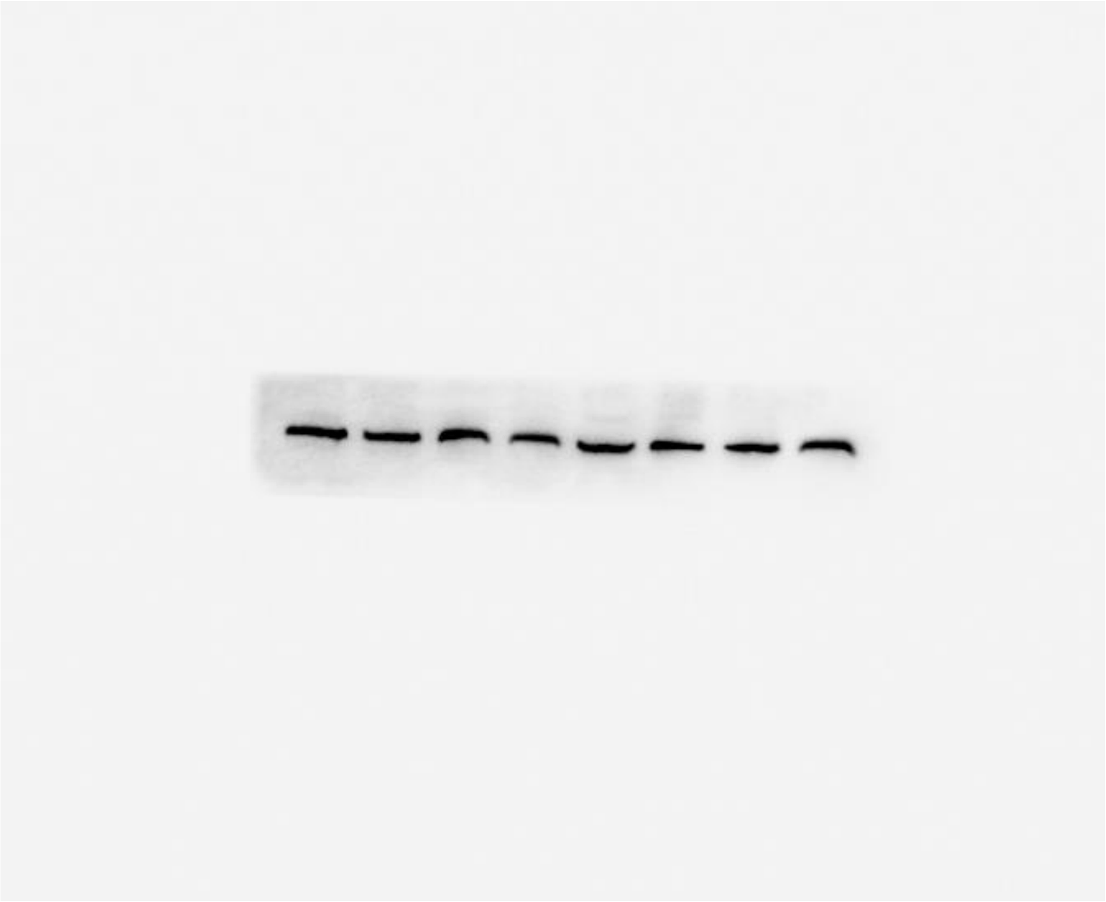

Fig 7C (IB: β-actin in SNU-638 cells)

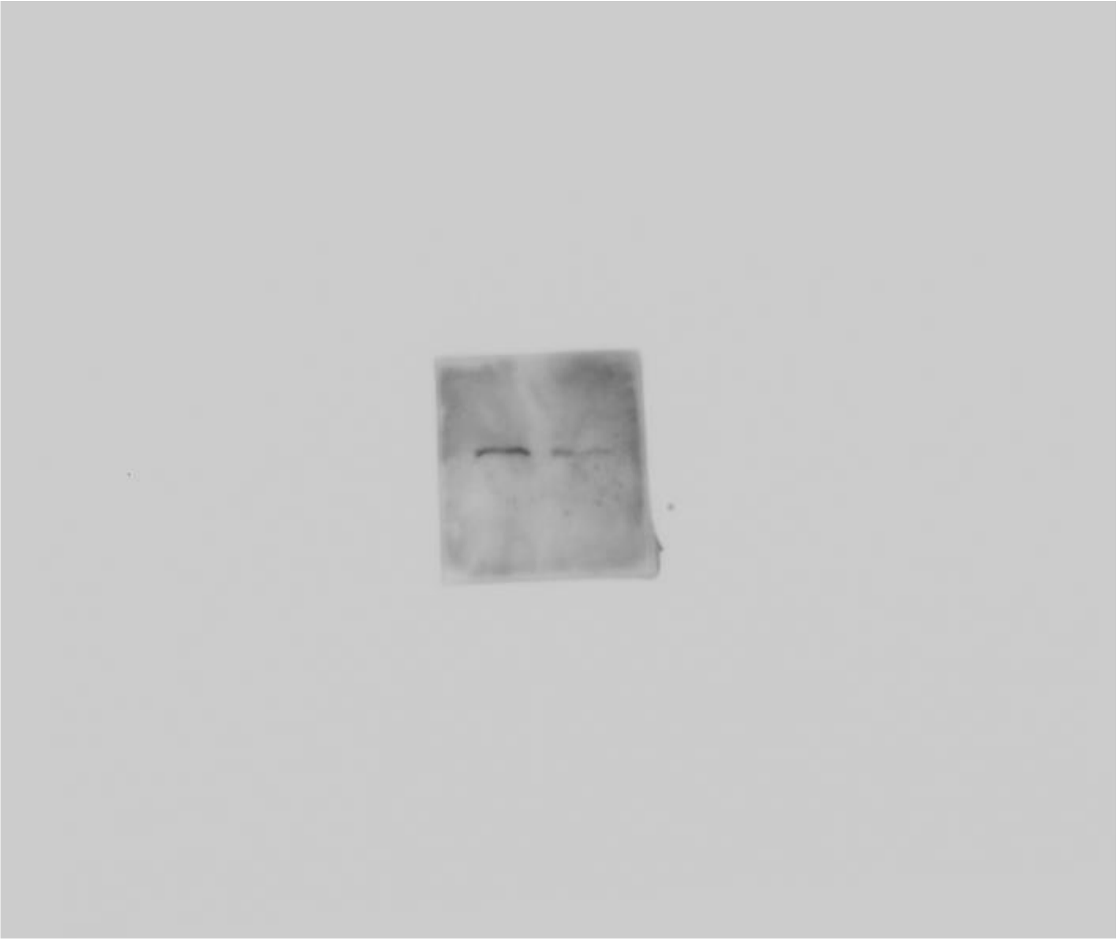

Fig 7E (IB: Ac-K in MKN-1 cells)

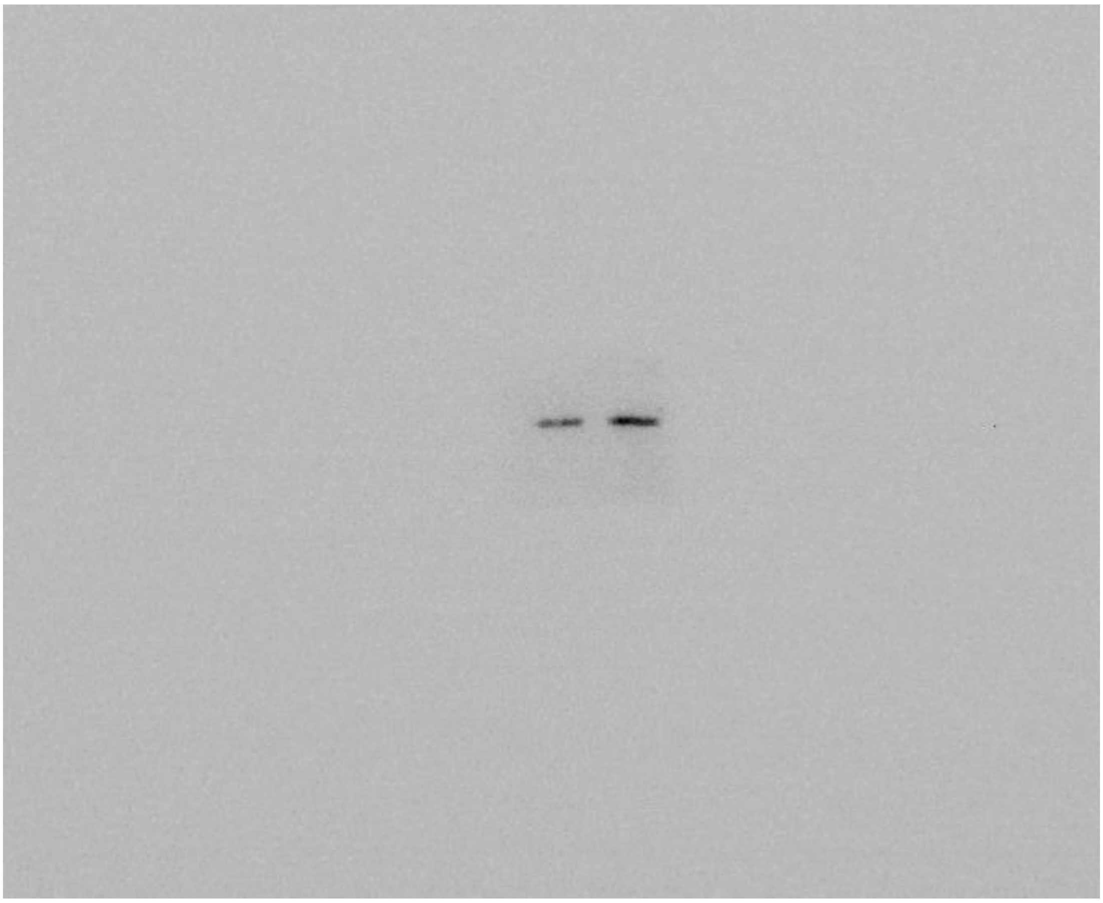

Fig 7E (IB: Ac-K in SNU-638 cells)

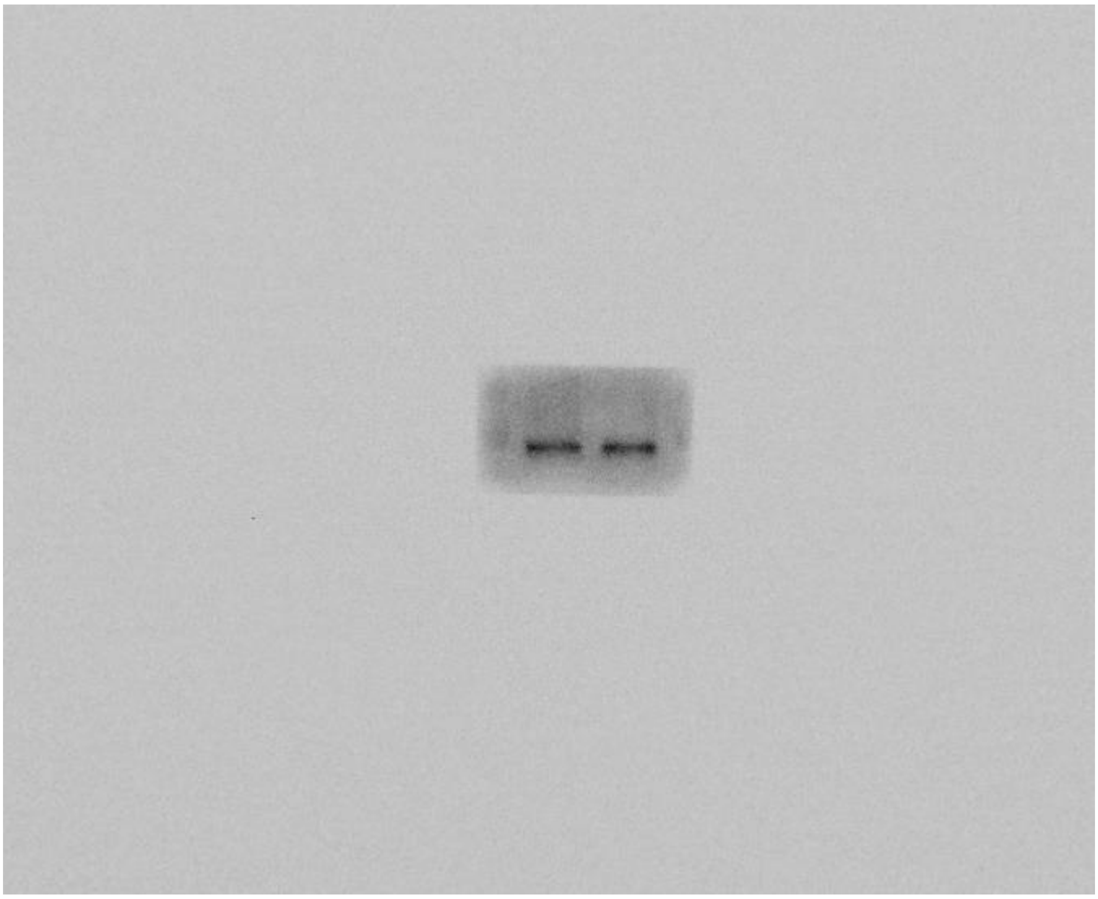

Fig 7E (IB: CPT2 in MKN-1 cells)

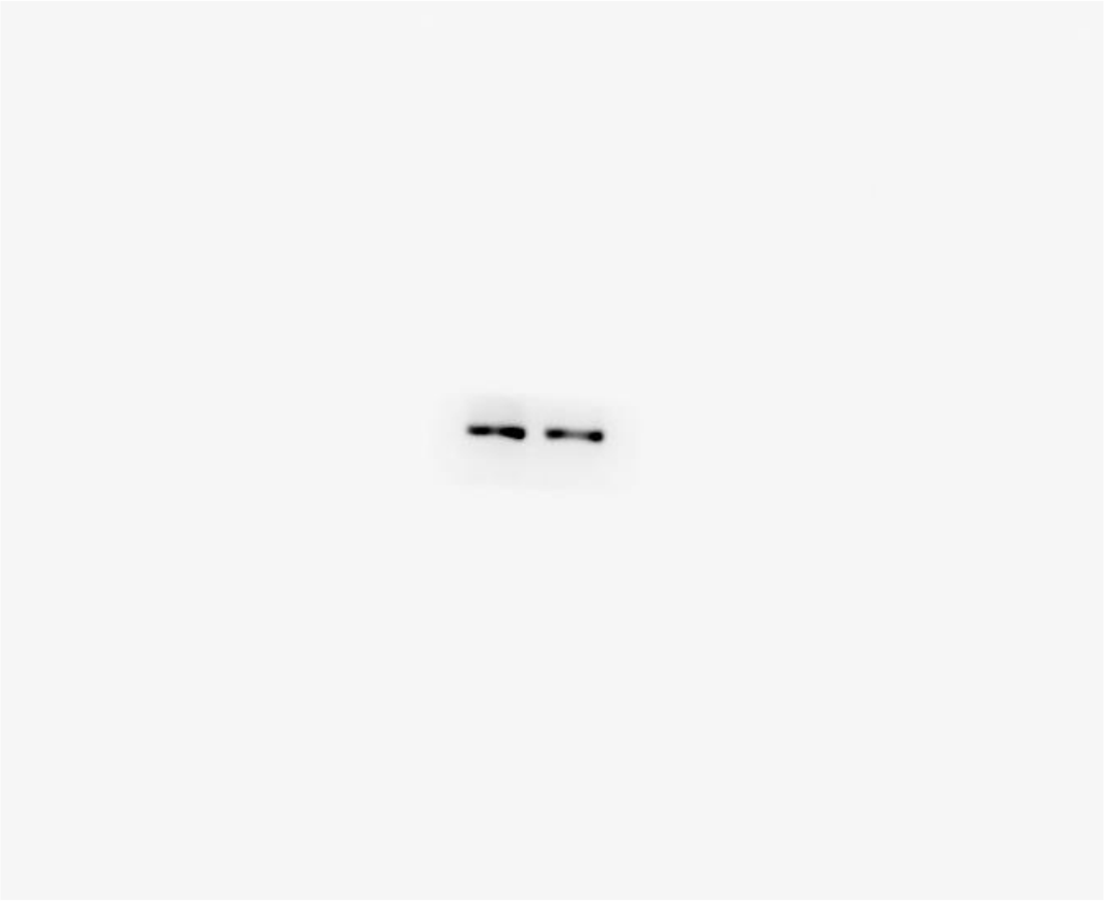

Fig 7E (IB: CPT2 in SNU-638 cells)

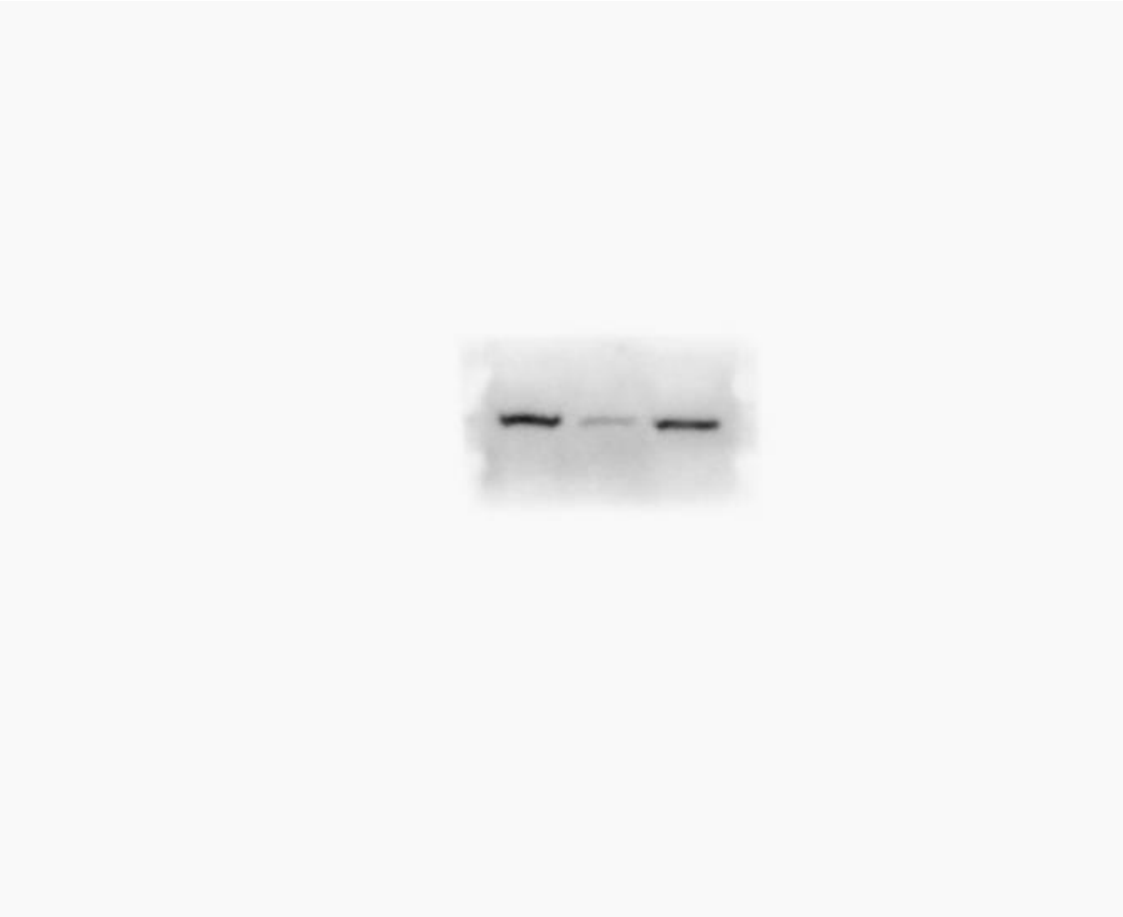

Fig 7F (IB: Ac-K in MKN-1 cells)

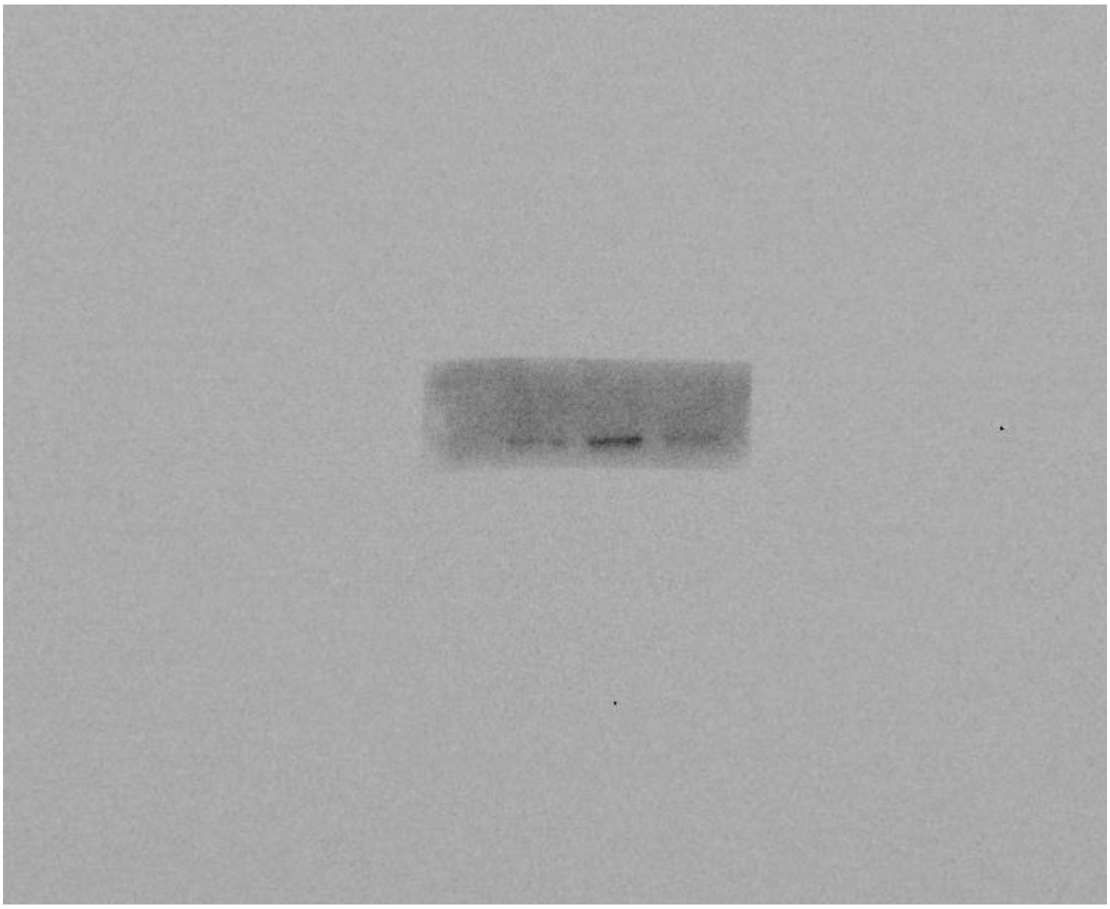

Fig 7F (IB: Ac-K in SNU-638 cells)

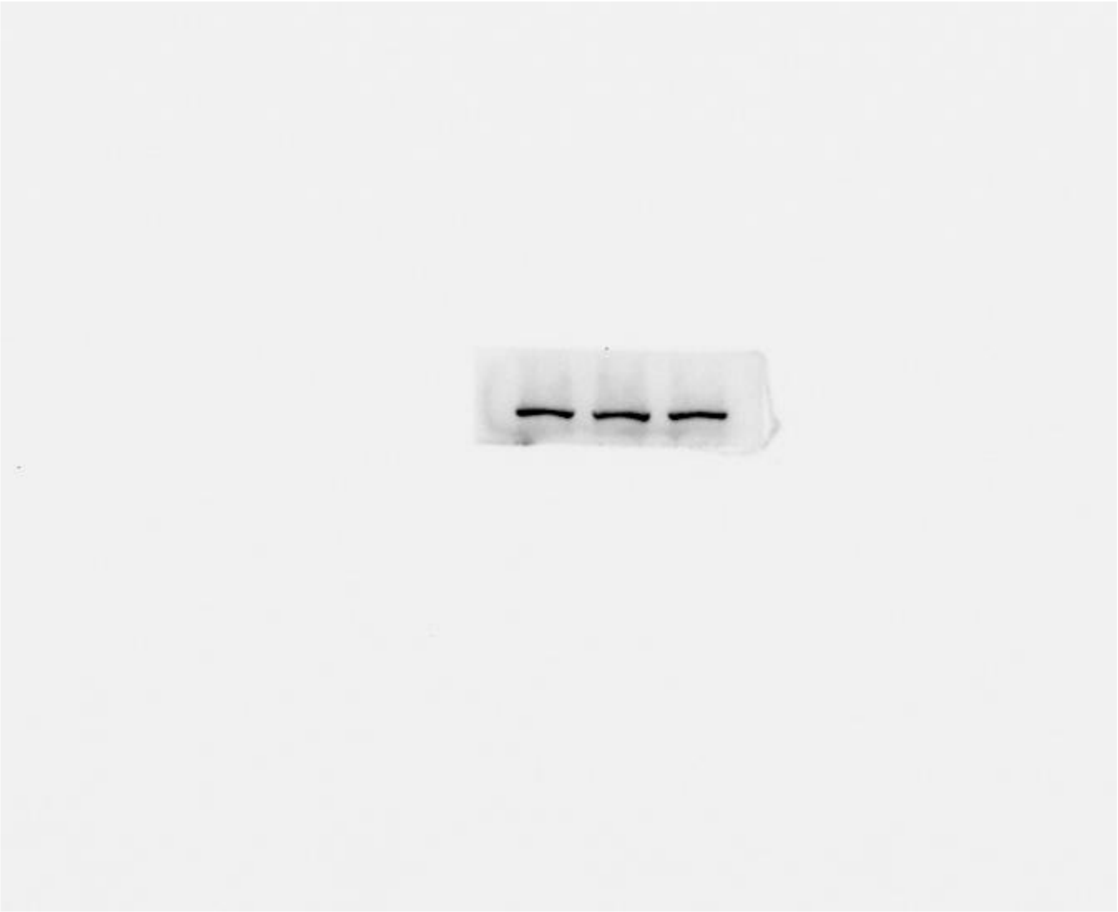

Fig 7F (IB: CPT2 in MKN-1 cells)

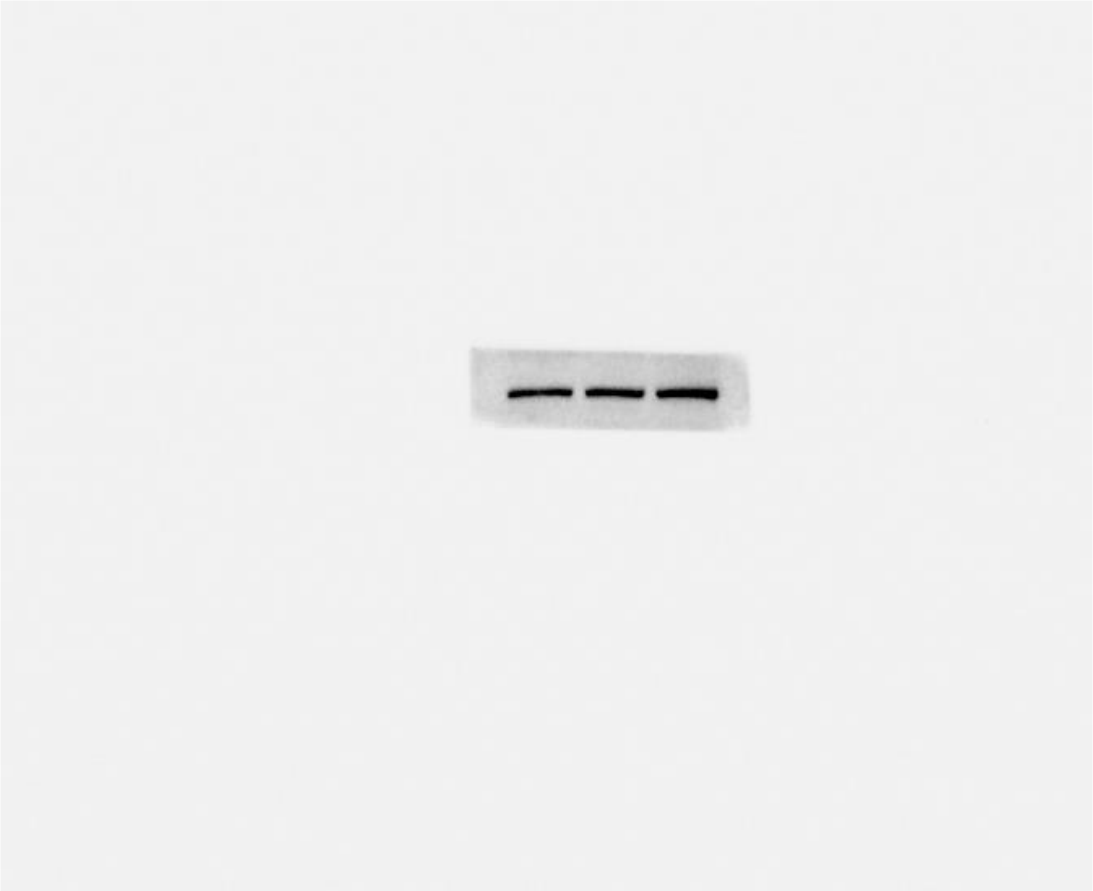

Fig 7F (IB: CPT2 in SNU-638 cells)

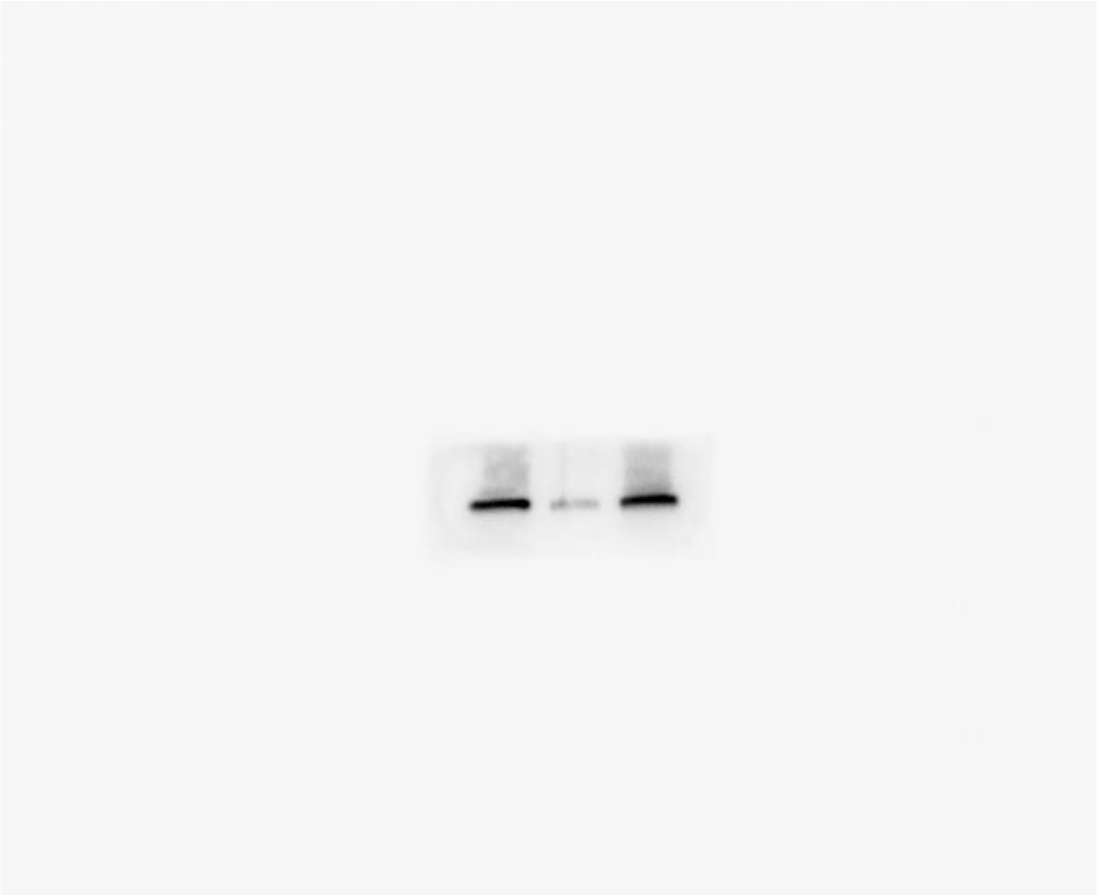

Fig 7G (IB: CPT2 in MKN-1 cells)

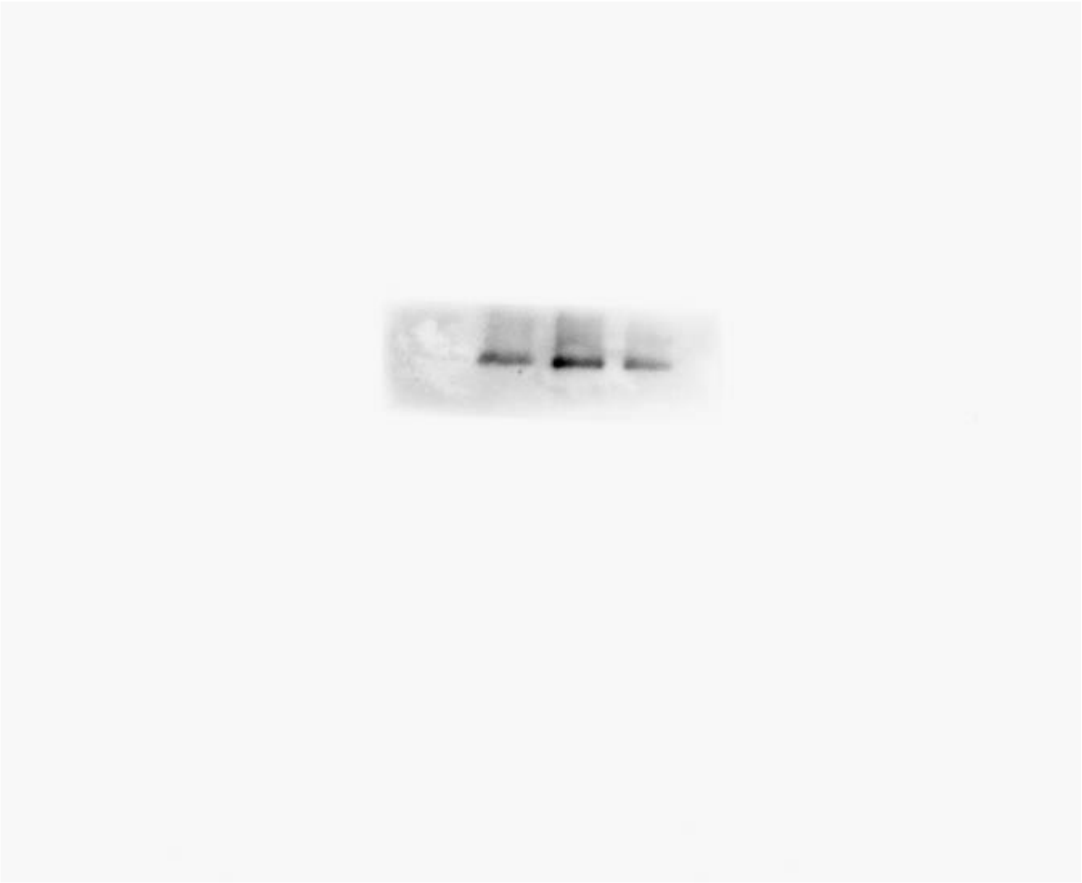

Fig 7G (IB: CPT2 in SNU-638 cells)

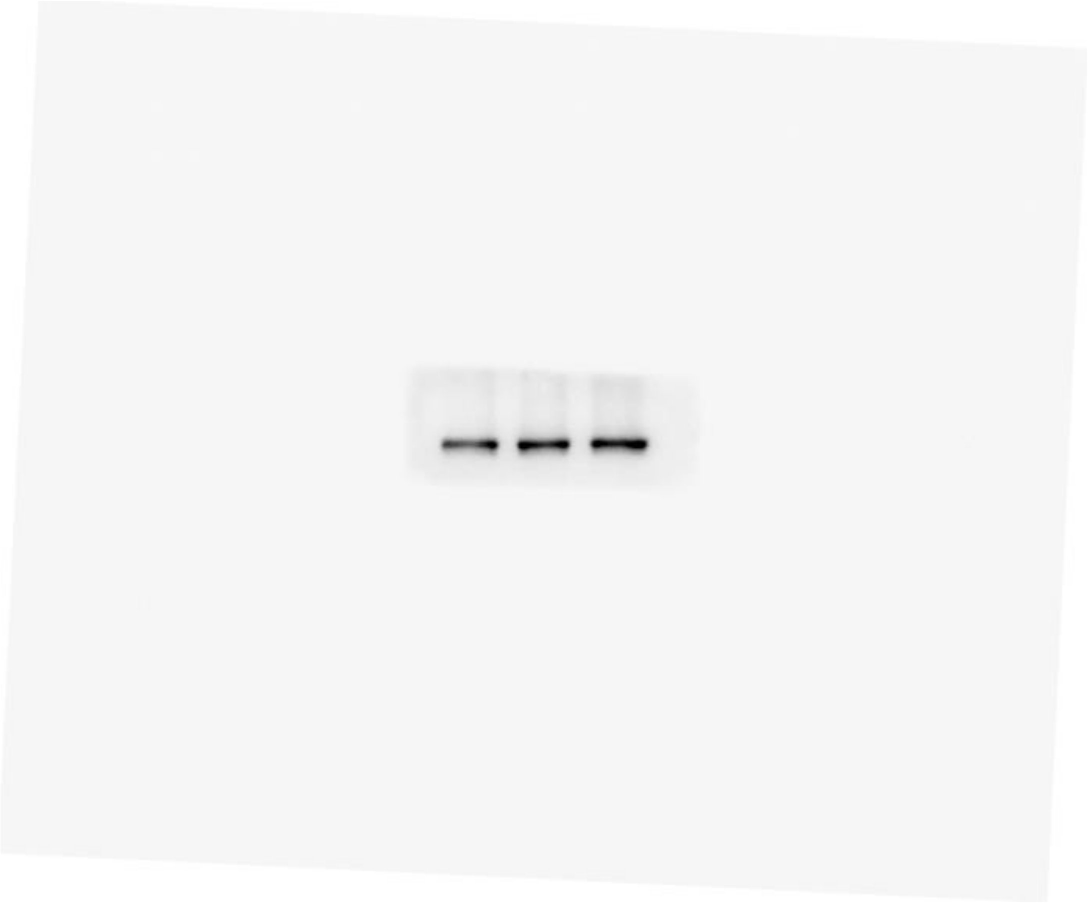

Fig 7G (IB: β-actin in MKN-1 cells)

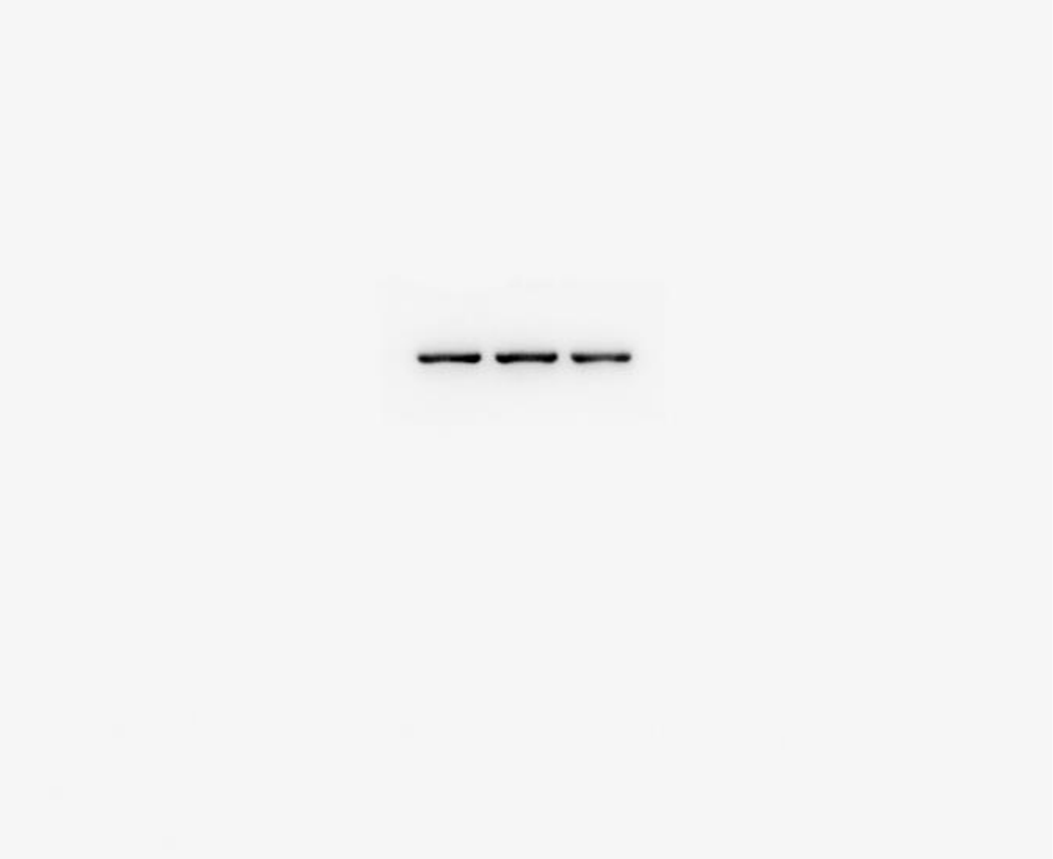
Fig 7G (IB: β-actin in SNU-638 cells)
